# Understanding the Impact of Contact-Induced Strain on the Electrical Performance of Monolayer WS_2_ Transistors

**DOI:** 10.1021/acs.nanolett.4c02616

**Published:** 2024-10-04

**Authors:** Lauren Hoang, Marc Jaikissoon, Çağıl Köroğlu, Zhepeng Zhang, Robert K. A. Bennett, Jung-Hwan Song, Jerry A. Yang, Jung-Soo Ko, Mark L. Brongersma, Krishna C. Saraswat, Eric Pop, Andrew J. Mannix

**Affiliations:** 1 Department of Electrical Engineering, Stanford University, Stanford, California 94305, United States; 2 Department of Materials Science & Engineering, Stanford University, Stanford, California 94305, United States; 3 Geballe Laboratory for Advanced Materials, Stanford University, Stanford, California 94305, United States; 4 Department of Applied Physics, Stanford University, Stanford, California 94305, United States; 5 Stanford Institute for Materials and Energy Sciences, SLAC National Accelerator Laboratory, Menlo Park, California 94025, United States

**Keywords:** 2D transistors, strain engineering, contact resistance, thermal annealing

## Abstract

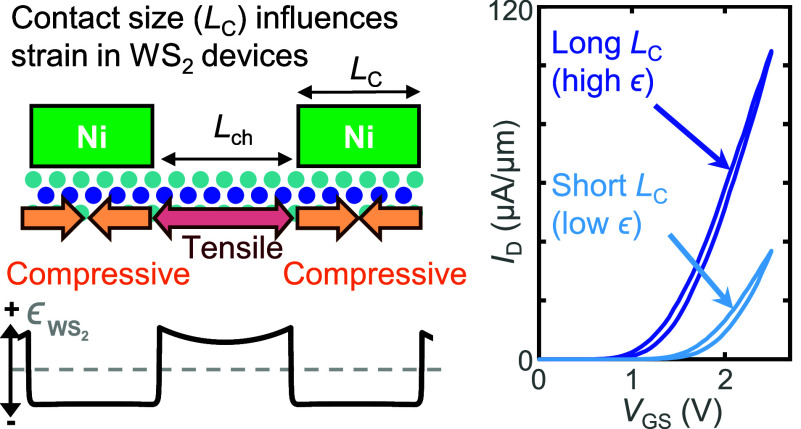

Two-dimensional (2D) electronics require low contact resistance (*R*_C_) to approach their fundamental limits. WS_2_ is a promising 2D semiconductor that is often paired with Ni contacts, but their operation is not well understood considering the nonideal alignment between the Ni work function and the WS_2_ conduction band. Here, we investigate the effects of contact size on nanoscale monolayer WS_2_ transistors and uncover that Ni contacts impart stress, which affects the WS_2_ device performance. The strain applied to the WS_2_ depends on contact size, where long (1 μm) contacts (*R*_C_ ≈ 1.7 kΩ·μm) show a 78% reduction in *R*_C_ compared to shorter (0.1 μm) contacts (*R*_C_ ≈ 7.8 kΩ·μm). We also find that thermal annealing can relax the WS_2_ strain in long-contact devices, increasing *R*_C_ to 8.5 kΩ·μm. These results reveal that thermo-mechanical phenomena can significantly influence 2D semiconductor–metal contacts, presenting opportunities to optimize device performance through nanofabrication and thermal budget.

Two-dimensional (2D) semiconductors, such as transition metal dichalcogenides (TMDs), have gained significant interest for next-generation electronics due to their atomically thin nature and good charge mobility in subnanometer films.^1,2^ Monolayer tungsten disulfide (WS_2_) has one of the highest predicted mobilities and largest band gaps of the TMDs, potentially enabling good low-power performance.^3−5^ However, further advances are limited by the high electrical contact resistance (*R*_C_) to WS_2_-based field-effect transistors (FETs).^6^ Several techniques have been shown to reduce the *R*_C_ to *n*-type TMDs, such as depositing semimetals (e.g., Bi and Sb),^7−9^ using low melting point metals (e.g., In and Sn),^10,11^ or transferred contacts,^12^ although many of these approaches are not considered industry-compatible. Furthermore, *n*-type contacts to WS_2_ lag behind MoS_2_ and more efforts are needed to investigate performance at reduced, submicron dimensions.

In principle, the *R*_C_ at the metal–semiconductor interface depends on: (1) the metal–semiconductor energy band alignment (including Fermi level pinning), where the metal work function (ϕ_m_) influences the Schottky contact barrier, and (2) the number of defects created during metal deposition, which tends to increase with the metal’s melting point^11^ or reactivity,^13,14^ thus causing additional Fermi level pinning. Nickel (Ni) is often used as the metal contact to *n*-type WS_2_,^15−19^ with the lowest reported *R*_C_ for Ni falling below 1 kΩ·μm at room temperature.^16^ In our devices, we find that Ni (Figure 1a, b) exhibits the lowest *R*_C_ compared to other metals such as Au, In, Sb (Figure S1). However, the origin of the low *R*_C_ for Ni on WS_2_ is not well understood and is in fact counterintuitive for two reasons: (1) Ni has a large work function (ϕ_Ni_ ≈ 5.15 eV),^20^ which is not well aligned to the conduction band of monolayer WS_2_ (Figure 1c); and (2) the high melting point of Ni (1455 °C) suggests that Ni contacts could produce more defects in the WS_2_ during physical vapor deposition.^20,21^

**Figure 1:**
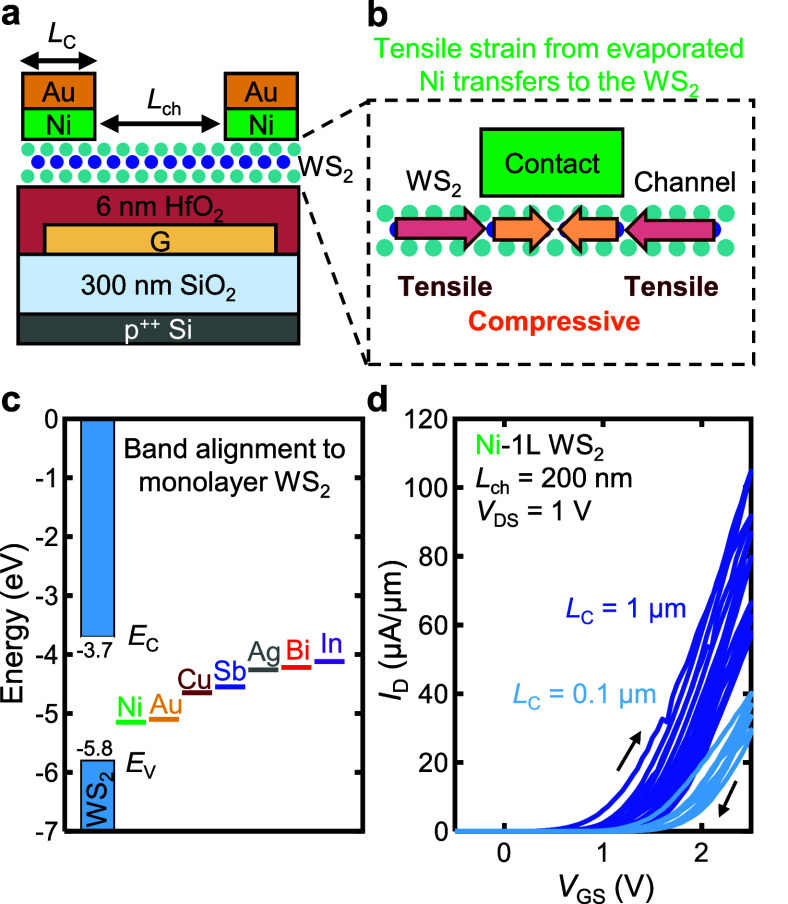
(a) Schematic of our monolayer (1L) WS_2_ devices on local back-gates with thin HfO_2_ and Ni contacts capped with Au. (b) Zoomed-in view of schematic in (a), showing strain in WS_2_ from Ni deposition. (c) The band gap of monolayer WS_2_ in approximate relation to the work function (ϕ_m_) of some bulk metals and semimetals. *E*_C_ denotes the conduction band edge and *E*_V_ denotes the valence band edge of 1L WS_2_. (d) Measured *I*_D_ vs *V*_GS_ curves for all *L*_ch_ = 200 nm devices with *L*_C_ = 1 μm (8 devices) and *L*_C_ = 0.1 μm (4 devices), showing clear *I*_D_ improvement with long contacts. Arrows denote the voltage sweep direction, showing clockwise hysteresis.

In contrast to the well-studied impact of metal work function alignment and metal-induced gap states upon *R*_C_, the mechanical effects of contacts are relatively underexplored. Thin metal films often contain high amounts of residual stress upon deposition, especially Ni.^22,23^ The band structure of 2D semiconductors is known to be sensitive to strain,^24,25^ and tensile strain has been recently shown to increase the electron mobility of monolayer MoS_2_ and WS_2_.^26−30^ Strain can be imparted by bending the substrate,^26−28^ by a capping layer,^29−33^ or at the contacts.^34,35^ In addition, the stress in such thin films can be modified by thermal treatment,^36^ which may impact the overall thermal budget for 2D transistor fabrication. Therefore, it is necessary to explore the potential role of strain in contact engineering, especially when depositing highly stressed metals like Ni (Figure 1a,b).^35^

In this work, we uncover the important role that strain plays at the Ni–WS_2_ interface and how it impacts the device transconductance (*g*_m_ = ∂*I*_D_/∂*V*_GS_) and *R*_C_. We observe that electron-beam deposited Ni applies high tensile stresses to the WS_2_ channel (Figure 1b). These stresses are approximately proportional to the contact length (*L*_C_), leading to ∼2.64× increase in on-state current with long contacts (*L*_C_ = 1 μm) compared to short contacts (*L*_C_ = 0.1 μm) for a channel length (*L*_ch_) of 50 nm (and ∼1.37× increase at *L*_ch_ = 1 μm). Long contacts also create significant strain near the WS_2_ contact region, resulting in 78% lower *R*_C_. However, we observe that thermal annealing causes relaxation of the strain in unencapsulated devices, resulting in nearly identical values of *R*_C_ for both long and short contact devices post-annealing. These observations highlight new strategies to boost device performance and emphasize the critical role of thermo-mechanical effects in evaluating different contact materials.

We studied monolayer WS_2_ devices in several geometries, predominantly using local back-gates with a low equivalent oxide thickness (EOT) insulator (see Methods). The local back-gates (Figure 1a) are patterned by lift-off from 2 nm/8 nm Ti/Pt (Pt on top) followed by thermal atomic layer deposition of 6 nm HfO_2_. The WS_2_ was grown separately on sapphire^37^ and transferred onto HfO_2_. WS_2_ channels were patterned by XeF_2_ etching, and transfer length method (TLM) structures with *L*_ch_ from 30 nm to 1 μm were defined using electron beam (e-beam) lithography. Ni (15 nm) capped with Au (20 nm) was deposited via e-beam evaporation at ∼10^–8^ Torr to form the contacts. The Ni/Au thin film exhibits a total stress of 160–175 MPa (Table S1). Electrical measurements were performed at 296 K under vacuum (∼10^–4^ Torr).

Because the stress applied by the contact increases with *L*_C_,^31,35^ contacts with *L*_C_ values of 0.1 and 1 μm were patterned to examine the effect of contact-applied stress on the device performance. As discussed later, the short *L*_C_ (0.1 μm) is more than twice as long as the current transfer length, indicating that current crowding should not affect device behavior. Figure 1d shows a comparison of measured transfer (*I*_D_ vs *V*_GS_) curves at *L*_ch_ = 200 nm between the short (*L*_C_ = 0.1 μm) and long (*L*_C_ = 1 μm) contact devices. Devices with longer contacts have higher drain current, more negative threshold voltage (*V*_T_), and increased *g*_m_. For such devices with *L*_ch_ = 200 nm, we find that increasing *L*_C_ (0.1 to 1 μm) doubles the median *I*_D,max_ (defined as the largest drain current *I*_D_ over the *V*_GS_ range applied) from 35.1 μA/μm to 70.2 μA/μm, shifts the median *V*_T_ negatively by −0.1 V (from 0.9 to 0.8 V), and increases the median peak *g*_m_ from 32.7 μS/μm to 54.3 μS/μm, at *V*_DS_ = 1 V.

To understand these changes, we performed finite-element analysis method simulations to reveal the impact of stressed metal contacts on the strain distribution along the WS_2_ channel, as a function of both *L*_C_ and *L*_ch_. Figure 2a shows the distribution of in-plane strain in WS_2_ devices with *L*_C_ = 1 μm and various *L*_ch_. For a long-channel device (*L*_ch_ = 1 μm), the tensile strain in the WS_2_ is highest next to the contact edge and decays toward the center of the channel. As the *L*_ch_ is reduced, the maximum WS_2_ tensile strain increases from 0.18% (*L*_ch_ = 1 μm) to 0.49% (*L*_ch_ = 30 nm) at the contact edge. The simulations are described in greater detail in Supporting Information Section 3, where it is also shown that the strain in WS_2_ is predominantly uniaxial, along the direction of current flow.

Figure 2b illustrates the strain in WS_2_ when *L*_C_ is reduced to 0.1 μm. Evidently, the maximum tensile strain is an order of magnitude lower than for *L*_C_ = 1 μm, ranging from 0.017% to 0.03% strain. This occurs because the stress in the contacts is determined primarily by the deposition conditions and is therefore independent of the contact geometry. The total (compressive) change in the *L*_C_ (the amount by which the contacts pull on the channel) is approximately proportional to the nominal *L*_C_. Thus, we find that both *L*_ch_ and *L*_C_ can significantly affect the tensile strain in the WS_2_ channel. The ratio between *L*_ch_ and *L*_C_ will dictate the overall strain profile: even at short *L*_C_, the strain in the channel will increase as *L*_ch_ is scaled down.

We used photoluminescence (PL) spectroscopy to validate the WS_2_ strain profile along the channel (see Supporting Information Section 4). Because tensile strain in WS_2_ reduces the direct band gap by lowering the conduction band, the PL peak is known to redshift with tensile strain.^38−40^ To probe the strain distribution in WS_2_ away from a contact edge, we map PL spectra along the WS_2_ with a pixel spacing of 200 nm. Figure 2c displays an optical image and the integrated intensity in the range 1.7–2.2 eV, to accurately determine the contact location (dark blue). Test devices were fabricated on 100 nm SiO_2_ (on Si) instead of metal local back-gates to accurately map the confocal PL response. The PL spectrum is fitted (with a weighted Gaussian–Lorentzian line shape) and the neutral A exciton peak is plotted with respect to the distance from the contact edge (Figure 2d). The A exciton peak of WS_2_ close to the contact edge is lowered by 20 meV, consistent with ∼0.5% tensile strain based on previous experimental work.^40^ The WS_2_ PL peak position reverts to its nominal value ∼1.5 μm away from the contact edge, illustrating an upper bound for the lateral decay length of the strain imparted by the stressed metal on WS_2_.^33^ We note that these measurements are constrained by the diffraction limit and PL cannot probe the WS_2_ under the metal contact. The signal arising from the nominal contact regions is due to the finite laser spot size, which collects some signal from the region outside the contacts.

We also performed PL mapping of a device with *L*_ch_ = 1 μm and *L*_C_ = 1 μm in Figure 2e,f. The PL peak in the channel is 30 meV lower than the nominal PL peak, corresponding to ∼0.7% strain induced in the material.^40^ The profile of the A exciton peak position in Figure 2f matches the expected strain profile obtained by simulations in Figure 2a both within the channel and outside the contacts, supporting our conclusion that the metal contacts induce strain in the WS_2_ (Figure S4a, b).

**Figure 2:**
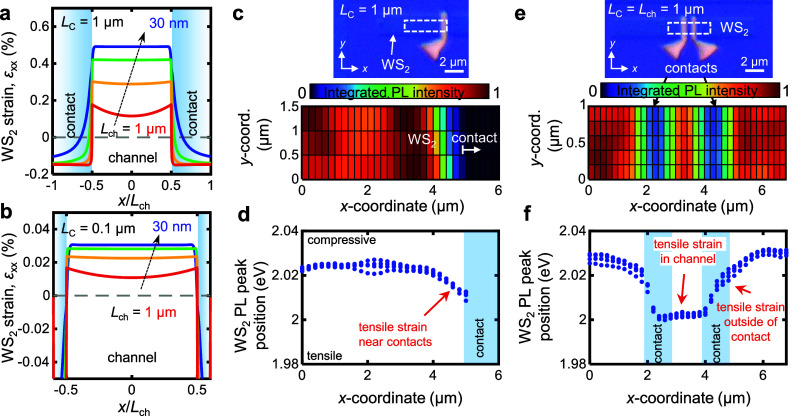
(a) Simulated horizontal strain profile along a device with contact length, *L*_C_ = 1 μm and channel length, *L*_ch_ = 1 μm, 300 nm, 100 nm, and 30 nm. The position *x* is normalized by *L*_ch_, for easier visualization. (b) Simulated horizontal strain profile along a device with *L*_C_ = 0.1 μm and *L*_ch_ = 1 μm, 300 nm, 100 nm, and 30 nm. (c) Integrated photoluminescence (PL) spectral intensity map away from a contact edge (on the right). (d) Measured A exciton peak in WS_2_ as a function of *x*-coordinate, for the map in (c). (e) Integrated PL spectral intensity map of a device with *L*_ch_ = 1 μm and *L*_C_ = 1 μm, demonstrating the position of the contacts (dark blue). (f) Measured A exciton peak of WS_2_ as a function of *x*-coordinate, for the map in (e). For panels (d, f), we note that the data plotted under the contact regions represent regions of WS_2_ just outside the contact edge due to the finite laser spot size (∼500 nm). WS_2_ directly under the contact cannot be probed due to the metal layers on top.

As seen in Figure 1d, the *g*_m_ and *I*_D_ are both strongly dependent on the contact and channel dimensions. The measured transfer curves in Figure 3a,b compare long contact (*L*_C_ = 1 μm) and short contact (*L*_C_ = 0.1 μm) devices for *L*_ch_ = 1 μm and 50 nm, respectively. At all *L*_ch_, we consistently observe that long contacts demonstrate higher *I*_D,max_. For the long-channels (*L*_ch_ = 1 μm), the on-state current (*I*_on_) at fixed overdrive voltage (*V*_ov_ = *V*_GS_ – *V*_T_) of 1.1 V has a median increase of 1.35× when switching from *L*_C_ = 0.1 μm to *L*_C_ = 1 μm (Figure 3a, c). In contrast, for short-channels (*L*_ch_ = 100 nm), the long-contact devices show a 2.67× increase in *I*_on_ (Figure 3b, c). Figure 3c summarizes the strong impact of *L*_C_ on the nonlinear relationship between *I*_on_ (at *V*_ov_ = 1.1 V) and *L*_ch_: namely, shorter channels display a greater increase of *I*_on_ when they have long contacts. This is consistent with the simulations in Figure 2a, where shorter channel devices exhibit a larger tensile strain in the WS_2_. Figure 3d plots the experimentally measured median peak *g*_m_ at each *L*_ch_, showing a clear increase in peak *g*_m_ for devices with long contacts. When *L*_ch_ is long (*L*_ch_ = 1 μm) the median peak *g*_m_ increases by 1.74× for the long contacts (i.e., higher strain). In devices with long *L*_ch_, which are dominated by the channel resistance *R*_ch_ (e.g., *R*_ch_ > 10 *R*_C_), it is reasonable to expect that *g*_m_ is mostly influenced by mobility.^41^ Thus, the increase in *g*_m_ is attributed to an increase in mobility in this long channel regime. This is consistent with recent studies which have found tensile strain to reduce intervalley scattering in the conduction band of monolayer MoS_2_ or WS_2_, leading to higher mobility.^26−29^

From our TLM structures, we also estimate the *R*_C_ with the two *L*_C_ values (Figure 3e), finding that long contacts lead to substantially lower *R*_C_ than short contacts. The longer *L*_C_ yields an *R*_*C*_ of 1.73 kΩ·μm at *V*_ov_ = 2.3 V for our best TLM structure. This is consistent with some of the best existing Ni contacts to monolayer WS_2_ in the literature, which range between 0.72 to 2.6 kΩ·μm (see Table S3).^15,18,42^ In terms of their nominal contact structure, these studies are equivalent to our “strained” long contact case, with Ni thicknesses >15 nm and *L*_C_ on the order of 1 μm. We note that the TLM extraction method for *R*_C_ assumes the same sheet resistance for all *L*_ch_, but this may exhibit some dependence on strain. However, ensuring the TLM data are taken at the same *V*_ov_ and having short channel devices (here, *L*_ch_ < 100 nm) in the TLM appears sufficient to estimate *R*_C_, because the short-channel devices are almost entirely contact-limited.

In contrast to the long contacts, our short contacts have *R*_C_ of 7.8 kΩ·μm at *V*_ov_ = 1.8 V. We note that our short *L*_C_ (0.1 μm) is comfortably greater than the estimated transfer length, *L*_T_ ≈ 37 nm (Supporting Information Section 6), indicating that the higher *R*_C_ is not due to current crowding,^2^ but is instead a result of the lower WS_2_ strain with the shorter contacts. The improvement in *R*_C_ likely stems from the higher concentration of electrons in the region next to the contacts. The high tensile strain near the contacts (Figure 2a) lowers the conduction band, increasing the electron concentration. Long contact devices also have lower *V*_T_ (by −0.27 V) (Figure S10b), which is consistent with greater tensile strain in the WS_2_ channel, lowering the conduction band edge and increasing the electron density in the channel and contacts. We can see that the difference in *L*_C_ significantly affects both *R*_ch_ and *R*_C_, with long contacts increasing *g*_m_ by 1.74× (which we attribute to enhanced mobility) and decreasing *R*_C_ by 78%.

Tensile strain in TMDs like WS_2_ lowers the conduction band edge,^27,43^ decreasing the Schottky barrier height (SBH) for electron injection at the contacts.^24,44,45^ We quantified the impact of contact-induced strain on SBH for the long (*L*_C_ = 1 μm) and short (*L*_C_ = 0.1 μm) contacts (Figure 3f), using temperature-dependent measurements (Figures S11, S12). The SBH decreases from ϕ_B_ ≈ 400 meV for short contacts to ϕ_B_ ≈ 170 meV for long contacts with greater strain (for the single devices plotted in Figure 3f). Comparing measurements for three long and three short contact devices reveals an average barrier height reduction of 170 meV from the strain induced by the long contacts. This substantial reduction illustrates that strain can modify the effective SBH at metal–2D interfaces independently from the metal work function. This dependence of *R*_C_ on strain and contact dimensions is often overlooked and may directly affect the apparent performance of various contacts reported in the literature. We expect these results to be qualitatively similar to other TMDs^25^ such as MoS_2_^27,35^ and WSe_2_^45^ due to their improvement in electron mobility with tensile strain.

**Figure 3:**
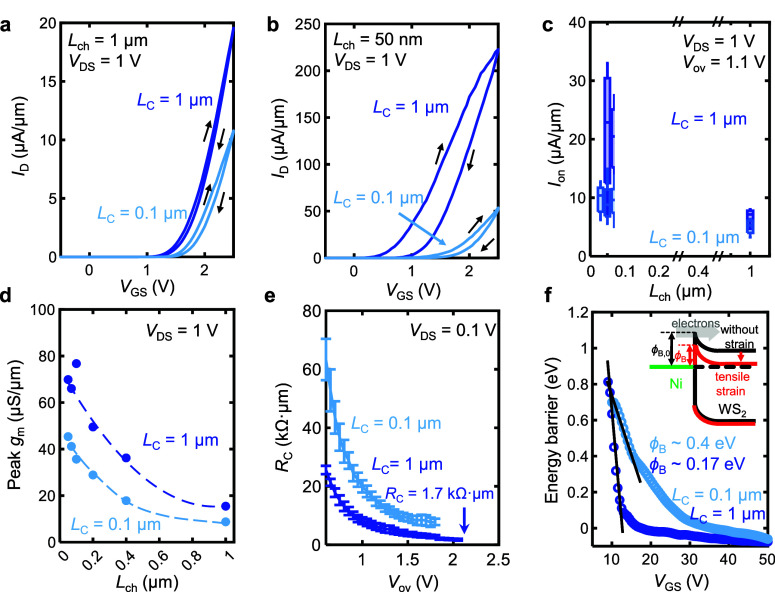
(a) *I*_D_ vs *V*_GS_ curves of a *L*_C_ = 1 μm and *L*_C_ = 0.1 μm device with *L*_ch_ = 1 μm. Arrows denote the voltage sweep direction, showing clockwise hysteresis. (b) *I*_D_ vs *V*_GS_ curves of a *L*_C_ = 1 μm and *L*_C_ = 0.1 μm device with *L*_ch_ = 50 nm. (c) On-state current (*I*_on_) at a fixed overdrive *V*_ov_ = 1.1 V versus *L*_ch_, comparing a total of 103 devices with *L*_C_ = 1 μm and *L*_C_ = 0.1 μm. (d) Peak transconductance (*g*_m_) vs *L*_ch_ for *L*_C_ = 1 μm and *L*_C_ = 0.1 μm, plotting the median device at each *L*_ch_. The dashed blue lines are qualitative guides to highlight the trend. (e) Estimated *R*_C_ for *L*_C_ = 1 μm and *L*_C_ = 0.1 μm, showing ∼5× reduction in *R*_C_ with long contacts (fitting the highest performing TLM). (f) Electron Schottky barrier height analysis for long contact and short contact devices, demonstrating a large decrease in barrier height (ϕ_B_) for long contact devices. Inset: Schematic of energy band diagram with strain experienced at the channel and near contacts.

Thermal processing is known to affect thin film stress and thus is expected to change the strain profile and electrical transport of the WS_2_ transistors. We investigate the impact of a 150 °C, 2-hour vacuum anneal on the Ni–WS_2_ device performance with respect to *L*_C_ and *L*_ch_. After annealing, the highly strained long contact devices (*L*_C_ = 1 μm) show decreased *I*_on_, an effect which is especially apparent for the short channel devices (Figure 4a, c). In contrast, the devices with short contacts (lower initial strain) showed negligible change in performance after annealing (Figure 4b, c). The largest decrease in *I*_on_ after annealing occurs for devices with long contacts and short channels (Figure 4c). Additionally, for the higher-strained, long-contact devices, annealing decreases the peak *g*_m_ from 17.1 μS/μm to 14.5 μS/μm for *L*_ch_ = 1 μm. This decrease in peak *g*_m_ for long channel devices is consistent with a loss of strain-induced mobility enhancement in the WS_2_ channel after annealing.

**Figure 4:**
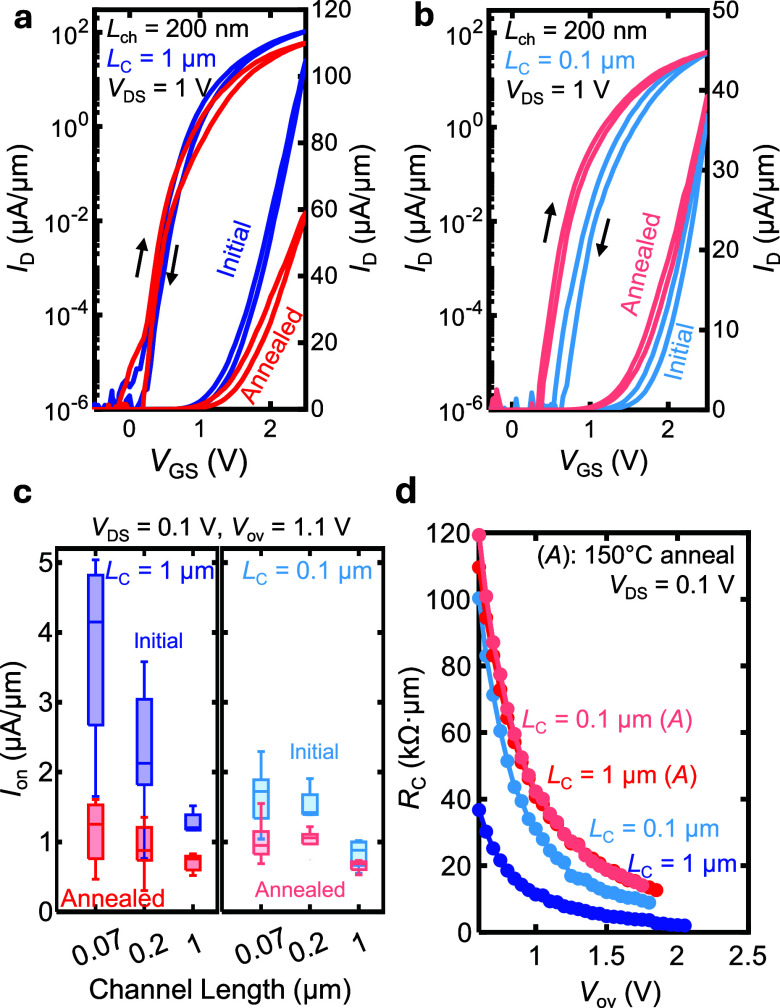
Contact length-dependent annealing effect on device performance. (a) Current vs gate voltage (*V*_DS_ = 1 V) of a *L*_C_ = 1 μm device before and after a 150 °C vacuum anneal. Arrows denote the voltage sweep direction, showing small clockwise hysteresis. (b) Current vs gate voltage (*V*_DS_ = 1 V) of a *L*_C_ = 0.1 μm device before and after a 150 °C vacuum anneal. (c) On-state current at a fixed overdrive *V*_ov_ = 1.1 V vs *L*_ch_ before and after annealing, for *L*_C_ = 1 μm (left) and *L*_C_ = 0.1 μm (right). Short channel, long contact devices show largest decrease in current. (d) *Median R*_C_ for *L*_C_ = 1 μm and *L*_C_ = 0.1 μm before and after annealing.

Figure 4d shows the increase in the *median R*_C_ due to annealing, for both the long contact devices (3.8 kΩ·μm to 14.1 kΩ·μm) and short contact devices (9.5 kΩ·μm to 14.1 kΩ·μm), at *V*_ov_ = 1.75 V. Thus, the value of *R*_C_ for long contact devices increased by ∼3.7× while the short contacts increased by only ∼1.5 ×. Furthermore, the final *R*_C_ after annealing for both the long contact and short contact devices are similar, both with a median TLM fit of 14.1 kΩ·μm at *V*_ov_ = 1.75 V. This suggests that the electrical properties of the WS_2_/Ni interface are rendered more similar following annealing. Evidently, the performance boost from strain induced by the long contacts is lost after annealing, which further supports the conclusion that strain is responsible for the improved *R*_C_ and *g*_m_. This also suggests that the relatively mild annealing condition of 150 °C is above the thermal budget for this combination of contact geometry and stress state in unencapsulated devices. However, the significant improvement in electronic properties provides strong motivation to preserve strain after annealing. We observe that encapsulating the devices with AlO_*x*_ successfully maintains the strain from the *L*_C_ = 1 μm contacts after annealing (Figure S13).

Our device observations are consistent with in-plane X-ray diffraction (XRD) analysis, which reveals changes in Ni/Au strain upon annealing (Figure S14). The Ni layer initially exhibited in-plane tensile strain (0.137%) which increased to 0.174% after annealing. Likewise, the initial compressive strain in the Au layer (−0.241%) was reduced after annealing (−0.127%). This net increase in tensile strain within the contact metals imparts a large stress to the WS_2_, causing the WS_2_ to exceed the traction limit and relax by slipping freely on HfO_2_.^33^ This aligns with simulation results in Figure S3, where weak mechanical coupling between the WS_2_ and HfO_2_ results in low strain in the WS_2_ channel.

We have considered other potential mechanisms that could explain the increase in *R*_C_ with annealing, such as diffusion^46^ or interfacial reactions.^14,47^ Ni has been shown to diffuse into MoS_2_ and increase *R*_C_ following annealing at 250–400 °C.^46^ Additionally, the oxidation of Ni has been proposed to explain increased *R*_C_ with annealing.^2^ However, these mechanisms do not account for the observation that the largest decrease in *I*_on_ occurs in the most highly strained devices (long contacts, short channel). Thus, we attribute the increased *R*_C_ after annealing to strain relaxation in the WS_2_.

In conclusion, we uncover that electron-beam evaporated Ni contacts impart tensile strain in a monolayer WS_2_ channel, ultimately reducing the *R*_C_. The effects of tensile stress in the Ni contact can be comparable in magnitude to the contributions from the work function and the metal–2D interfacial quality. WS_2_ devices with long contacts (1 μm) have a best (median) *R*_C_ of 1.73 (2.00) kΩ·μm which worsens for short (0.1 μm) contacts to 7.8 (8.9) kΩ·μm. We also demonstrate that thermal annealing affects contact-induced strain, which depends on the contact length, *L*_C_.

Considering the key role of *R*_C_ as a limiting factor in device performance and the numerous recent studies of contacts in 2D transistors, these results highlight the importance of carefully examining metal strain effects to determine the origin of changes in *R*_C_. This is especially important when considering the implications for *L*_C_ scaling: many contemporary studies use contacts long enough to cause strain effects, yet future nanoscale devices must use short contacts. The sensitivity of *R*_C_ and mobility to annealing conditions, resulting from strain relaxation, also suggests that strict attention to thermal budget or strain stabilization is required.

## Methods

### WS_2_ Growth

Monolayer WS_2_ was grown on SiO_2_/Si substrates and sapphire by chemical vapor deposition (CVD) using diethyl sulfide (DES) and ammonium metatungstate (AMT) precursors.^37^ 0.6 g AMT and 0.1 g potassium hydroxide were dissolved in 30 mL deionized water and dip-coated on the edges of the substrate. For the sapphire (SiO_2_) substrate, the substrate is annealed in the furnace at 775 °C for 6 h with a DES flow rate of 0.05 sccm (0.12 sccm). N_2_ and H_2_ were used as carrier gases during the growth. Representative Raman and PL spectra of the WS_2_ films are shown in a previous study by Zhang et al.^37^

### Device Fabrication and Electrical Measurements

Monolayer WS_2_ was grown on sapphire and then transferred onto local back-gates covered by 6 nm HfO_2_. Device fabrication and transfer details have been described in depth previously.^48^ In summary, the local back gates were defined by lift-off of 2 nm/8 nm Ti/Pt followed by the HfO_2_ gate dielectric by thermal atomic layer deposition at 200 °C. Coarse contact pads were then defined by lift-off of 2/20 nm Ti/Pt. Polystyrene (PS) was spin-coated on top of the WS_2_ and then transferred using NaOH, with thorough rinsing in DI water. An O_2_ plasma treatment (100 W, 1 min) of the HfO_2_ dielectric was done before transferring the PS/WS_2_ film to modify the substrate’s surface energy. PS was removed in toluene, then a vacuum anneal (200 °C, 2 h, ∼10^–6^ Torr) was performed to promote adhesion. After the transfer, channel definition was done using XeF_2_ etching. Electron beam lithography was used to pattern the fine contacts. Ni/Au (15/20 nm) were e-beam evaporated at ∼10^–8^ Torr. Electrical measurements were performed at 296 K in a Janis ST-100 vacuum probe station at ∼10^–4^ Torr, using a Keithley 4200 semiconductor parameter analyzer.

For contact resistance (*R*_C_) extraction, the transfer length method (TLM) was used. In a two-terminal device, the major components are the *R*_C_ and the channel resistance (*R*_ch_). The total resistance in kΩ·μm (normalized by the channel width) can be expressed as *R*_TOT_*=* 2*R*_C_ + *R*_ch_*=* 2*R*_C_ + *R*_sh_*L*_ch_, where *R*_sh_ is the sheet resistance of the channel. The *R*_C_ is evaluated by plotting *R*_TOT_ versus *L*_ch_ and the *y*-intercept at *L*_ch_ = 0 gives the resultant 2*R*_C_. The *R*_C_ is extracted for each gate overdrive *V*_ov_ = *V*_GS_ – *V*_T_, with *V*_T_ from the constant-current method at *I*_D_ = 10^–2^ μA/μm.^49^

## Supporting Information Available

Device fabrication process, stress characterization, finite element analysis information, photoluminescence spectroscopy measurement and peak fitting, literature benchmarking, transfer length determination, transconductance threshold voltage extractions, Schottky barrier height extraction, density functional theory simulations, effect of annealing on Ni–WS_2_ devices on SiO_2_, and X-ray diffraction (PDF)
