# Supplementary material for: Understanding the Impact of Contact-Induced Strain on the Electrical Performance of Monolayer WS2 Transistors
Source: Nano Lett. 2024 Oct 4;24(41):12768–74. doi: 10.1021/acs.nanolett.4c02616 (PMC11488502; doi:10.1021/acs.nanolett.4c02616)
Supplement: Supplementary file 1 — nl4c02616_si_001.pdf [file nl4c02616_si_001.pdf]

Supporting Information

Understanding the Impact of Contact-Induced Strain on the Electrical Performance of Monolayer WS_2 Transistors

Lauren Hoang[1], Marc Jaikissoon[1], Çağıl Köroğlu[1], Zhepeng Zhang[2], Robert K. A. Bennett[1], Jung-Hwan Song[3], Jerry A. Yang[1], Jung-Soo Ko[1], Mark L. Brongersma[3], Krishna C. Saraswat[1,2], Eric Pop[1,2,4], and Andrew J. Mannix[2,5,*]

[1]*Department of Electrical Engineering, Stanford University, Stanford, CA 94305, USA*

[2]*Department of Materials Science & Engineering, Stanford University, Stanford, CA 94305, USA*

[3]*Geballe Laboratory for Advanced Materials, Stanford University, Stanford, CA 94305, USA*

[4]*Department of Applied Physics, Stanford University, Stanford, CA 94305, USA*

[5]*Stanford Institute for Materials and Energy Sciences, SLAC National Accelerator Laboratory, Menlo Park, CA 94025, USA*

1. Global Back-gated Device Fabrication and *n*-type Contact Resistance to WS_2

Monolayer WS_2 was grown by CVD directly onto 100 nm of SiO_2 with a Si (p^{++}) substrate serving as the global back-gate (**Figure S1a**). Discrete triangular monolayer WS_2 flakes were identified and used for the devices. XeF_2 was used to etch the WS_2 into well-defined channels, and transfer length method (TLM) structures with contact length L_C = 1.5 μm and varying channel lengths (L_{ch} = 100 nm–1 μm) were defined by electron beam (e-beam) lithography. We e-beam evaporate various contacts (Ni, Au, In, Sb) at ~10^{-8} Torr to examine the effects of metal contact on R_C. The exact metal splits used here were: Ni/Au (20/35 nm), Au (55 nm), In/Au (10/45 nm), Sb/Au (20/25 nm). Devices were measured in vacuum at 296 K before and after a 250 °C, 2 hour vacuum anneal. Annealing is commonly used to evaporate adsorbates off the TMD surface, resulting in lower hysteresis and improved current drive.

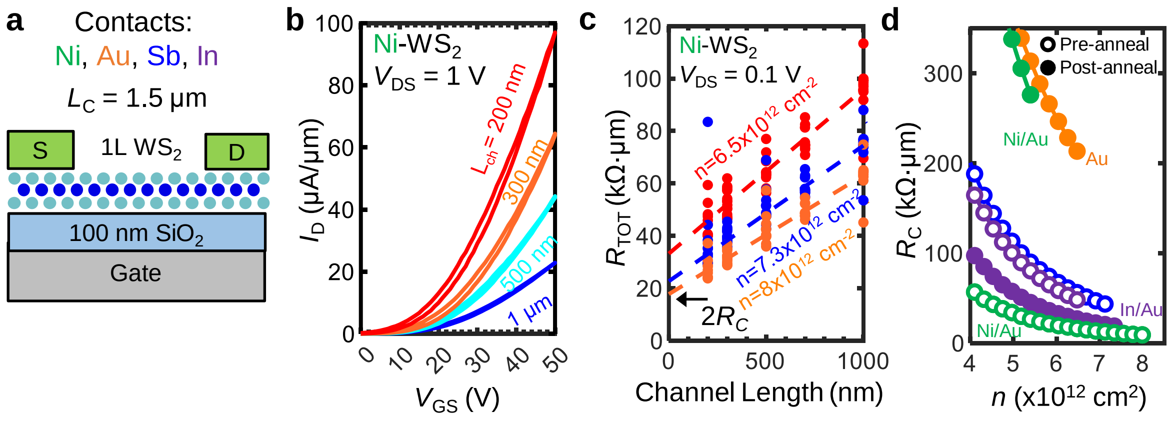

Figure S1. (a) WS_2 device schematic on 100 nm SiO_2. All devices were patterned with contact length L_C = 1.5 μm. **(b)** Current vs. gate voltage (V_{DS} = 1 V) of a L_{ch} = 1 μm, 500 nm, 300 nm and 200 nm channel for Ni contacts. **(c)** Total device resistance R_{TOT} vs. L_{ch} measured by TLM, at various carrier densities n, showing R_C extraction at the y-intercept. **(d)** Extracted R_C for various contact metals to WS_2, with Ni achieving the lowest contact resistance. Filled circles indicate data after 250°C annealing.

Figure S1b shows measured drain current (I_D) vs. gate voltage (V_{GS}) for Ni-contacted WS_2 with different channel lengths, before any annealing. The carrier density is obtained from the gate overdrive $V_{GS} - V_T$, where V_T was extracted by the constant-current method with a threshold current of $I_D = 10^{-2}$ μA/μm (IRDS high-performance limit). **Figure S1c** shows good linear fits to the total device resistance normalized by width (R_{TOT}) vs. L_{ch}. The vertical intercept of the linear fit yields the total contact resistance ($2R_C$). **Figure S1d** shows the R_C before and after annealing for all the contact metals (Ni, Au, Sb, In), with the lowest contact resistance obtained for Ni contacts pre-anneal. We uncover that the R_C significantly changes after annealing and is dependent on the contact metal. While Au has proven to be a good contact to MoS_2,[1] this was not the case for WS_2 and resulted in the highest R_C of the metals tested. Improvements in performance were seen when switching to Sb, In and Ni contacts. Sb contacts were not annealed due to thermal stability concerns.[2] While the In contacts improved with annealing, the Ni R_C worsened. Ni has been used as a standard contact for *n*-type WS_2 and is reaffirmed to be a good contact to WS_2, especially without any annealing.

2. Wafer-scale Ni/Au Stress Characterization

15/20 nm of Ni/Au was electron-beam evaporated at ~10^{-8} Torr onto a 350 µm thick (100) Si wafer terminated by native oxide. The wafer curvature was measured before metal deposition, after metal deposition, and after a 2 h, 150 °C vacuum anneal. Using the Stoney equation,[3] the film stress in the Ni/Au after e-beam deposition was found to be 160–175 MPa tensile. After the 150 °C 2 hour vacuum anneal, the film stress increased to 210–220 MPa tensile stress. Thin film force is given by $F = \sigma t$, where σ is the thin film stress and t is the thickness. The values found are summarized in **Table S1**. These values are consistent with the XRD data in **Supporting Information Section 10**, where the tensile in-plane strain increased with annealing.

Table S1: Extracted thin film stress from metal contact deposition.

**Metal stack, nm**	**Anneal?**	**Stress σ, MPa**	**Thin film force, N/m**
Ni/Au, 15/20	No	160–175	6
Ni/Au 15/20	Yes (150 °C, 2 hours)	210–220	7.5
Ni, 15	No	375– 410	6

3. Finite Element Analysis Simulations

Two-dimensional (2D) and three-dimensional (3D) stress simulations were performed for back-gated (BG) transistors, assuming linear elasticity. Multi-scale finite-element method simulations of the entire sample (including the transistor and the entire silicon substrate) confirmed that strains due to substrate bowing were negligible in comparison to those induced by the stressed contacts. To reduce simulation complexity, it thus suffices to simulate a smaller domain around the transistor with a fixed boundary condition at the bottom of a thinner section of substrate, with no appreciable errors in stress and strain distributions. In addition, we confirmed through 3D simulations that the WS_2 strain in the transistor width direction is small (WS_2 strain is essentially uniaxial and along the direction of current flow), as illustrated by **Figure S2**. Consequently, 2D simulations accurately capture the uniaxial stress and strain distributions in our devices, so the results presented in this work are generated using 2D simulations.

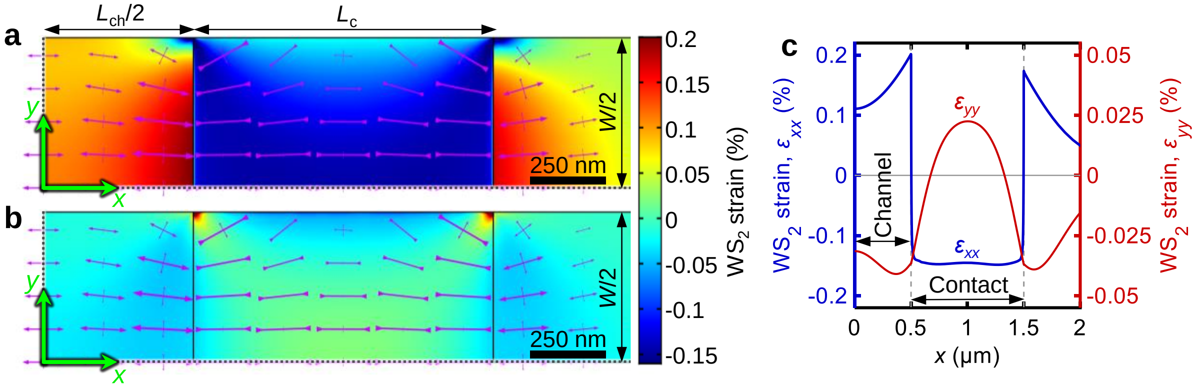

Figure S2. (a, b) The distributions of lengthwise in-plane strain [ε_{xx} given in (a)] and widthwise in-plane strain [ε_{yy} given in (b)] in WS_2 in a transistor with $L_{ch} = L_C = 1$ μm, viewed from above. The strain is induced by isotropic in-plane stress built into the contact electrodes. Only a quarter of the device is shown: the bottom left corner corresponds to the center of the device, with the dashed lines indicating the two symmetry planes. The magenta arrows indicate the principal strain directions at each point, showing that the strain is predominantly in the direction of current flow (i.e. along the *x*-axis) and hence approximately uniaxial, except very close to the edge of the channel. **(c)** WS_2 strains ε_{xx} (left axis) and ε_{yy} (right axis) along positive *x*-axis of the same device showing the widthwise strain is small (note the right axis is 4× smaller in scale).

In the finite element analysis, strain profiles are plotted against the *x*-coordinates of the unrelaxed geometry to consistently track and compare the same specific physical points across simulations. Each data point corresponds to a node, which retains its identity before and after relaxation. This method simplifies comparing strain profiles, as the *x*-coordinate always refers to the same physical point, despite deformation.

Since the stress in Au is small compared to Ni in the 15 nm Ni/20 nm Au stack, and given that Au is more mechanically compliant than Ni (i.e. lower in Young's modulus), we assume that the Au layer does not have a significant effect on the strain profile in WS_2 (at least before annealing) and is not included in the simulations. An in-plane tensile "initial stress" (the stress before the geometry is allowed to relax) of 400 MPa was assumed in Ni, equivalent to the 170 MPa of stress (6 N/m film force) of the thicker Ni/Au stack extracted from substrate bowing measurements. The isotropic elastic properties assumed for the materials other than WS_2 are summarized in **Table S2**. In contrast, WS_2 is only transversely isotropic (i.e. isotropic in-plane), and thus is described by an anisotropic stiffness tensor. Its elastic properties were taken from Li *et al.*,[4] and can be summarized as $E_{xx} = E_{yy} = 252$ GPa, $E_{zz} =$

50 GPa, $G_{xz} = G_{yz} = 26$ GPa, $\nu_{xy} = \nu_{yx} = 0.18$ and $\nu_{xz} = \nu_{yz} = 0.10$. Here, x and y correspond to the in-plane directions and z to the cross-plane direction, E denotes Young's modulus, G denotes shear modulus and ν_{ij} denotes Poisson's ratio for loading along i and transverse direction j. The remaining elastic properties can be determined from these, e.g. $\nu_{zx} = (E_{zz}/E_{xx})\nu_{xz}$ and $G_{xy} = E_{xx}/[2(1 + \nu_{xy})]$. Any strain imparted on WS_2 during growth or transfer is not included in these simulations, so the simulated WS_2 strain results should be interpreted as relative to WS_2 as-transferred.

Table S2: Young's moduli and Poisson's ratios assumed for materials except for WS_2.

	Si	SiO_2	Pt	HfO_2[5,6]	Ni
**Young's modulus (GPa)**	170	70	400	170	70
**Poisson's ratio**	0.28	0.17	0.22	0.25	0.44

Due to poor adhesion provided by the weak van der Waals forces, it is possible for there to be some amount of relative displacement (and even slipping) between WS_2 and adjacent materials, especially between WS_2 and the underlying HfO_2.[7] A shear-lag model has previously been applied to 2D materials to capture this effect.[7–9] To implement it in simulations, we apply a boundary condition between WS_2 and HfO_2 that is equivalent to a very thin elastic adhesive, which acts as a bed of springs opposing the relative lateral displacement of WS_2 on HfO_2. The shear stiffness κ of the adhesive is equal to the lateral traction (force per unit area) per unit displacement, and quantifies how much lateral force it takes for WS_2 to slide on HfO_2. If κ is low enough such that WS_2 is sufficiently decoupled from the layer underneath, we may express the characteristic strain decay length[7,10] (also known as the shear-lag length) as $\lambda \cong (E't/\kappa)^{1/2}$, where t is the WS_2 thickness, $E' = E/(1 - \nu^2)$ is the effective in-plane Young's modulus of WS_2 constrained such that there is no strain along its width (also known as 2D P-wave modulus), and E and ν are WS_2's in-plane Young's modulus and Poisson's ratio, respectively.

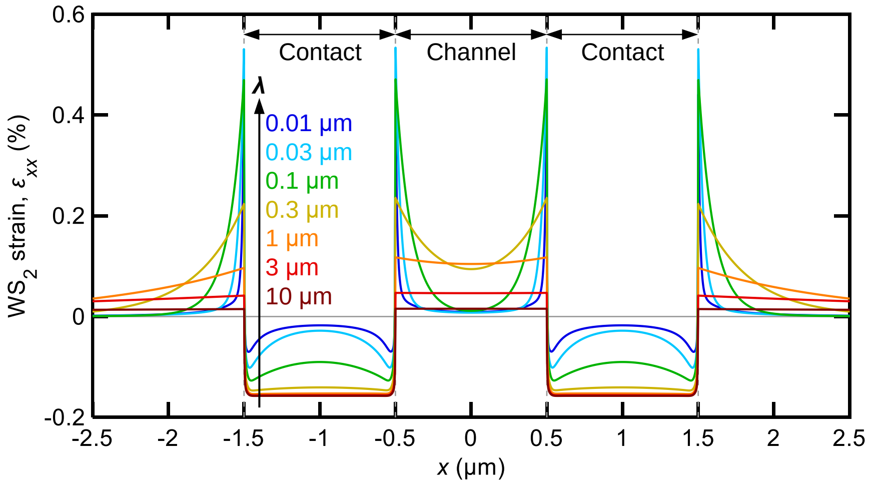

Figure S3. The contact-induced in-plane strain distribution in WS_2 in a transistor with $L_{ch} = L_C = 1$ μm, for several different values of characteristic strain decay length λ, which is approximately inversely proportional to the square root of the restoring shear "spring constant" which couples WS_2 to the underlying HfO_2. Weaker coupling (easier sliding) generally yields a lower peak strain and a more uniform strain distribution in the channel. $\lambda = 0.5$ μm is assumed in the other simulation results presented in this work.

Values of λ ranging from about 0.3 μm to 15 μm have been reported for various monolayer 2D materials on bulk substrates.[7,9,11–14] For our WS_2 films on SiO_2, photoluminescence measurement results in **Figure 2c-d** suggest that λ is comparable to 0.5 μm, so we have chosen $\lambda = 0.5$ μm (corresponding to $\kappa = 650$ MPa/μm) for our simulations of WS_2 on HfO_2 as a reasonable estimate. This value is also close to what has been reported for WS_2 on a polymer substrate.[12] The impact of other possible values of κ (and hence λ) on the strain profile in a transistor with $L_{ch} = L_C = 1$ μm is explored in **Figure S3.** It can be seen that for very high κ (no slip), only the region of the channel within ~50 nm of the contacts have appreciable strain. Much more of the channel is strained if WS_2 is able to slide more easily on HfO_2, and the entire channel is strained if λ is comparable to or greater than L_{ch}. For very small κ ($\lambda \gg L_{ch}$), the compressive strain under the contract is balanced by a small strain distributed mostly in a long region beyond the contacts ($x > 1.5$ μm in **Figure S3**), so the channel strain is likewise small.

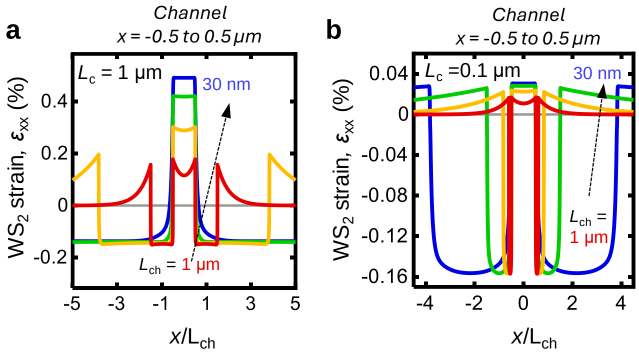

Figure S4. (a) Simulated horizontal strain profile along a device with $L_C = 1$ μm and $L_{ch} = 1$ μm, 300 nm, 100 nm, and 30 nm. The x position is normalized by L_{ch}, for easier visualization of different channel lengths. **(b)** Simulated horizontal strain profile along a device with $L_C = 0.1$ μm and $L_{ch} = 1$ μm, 300 nm, 100 nm, and 30 nm. Similar to panel a), the x position is normalized by L_{ch}.

Figure S4a,b demonstrates the compressive strain under the contacts, which appears to be relatively similar in magnitude, regardless of channel length. At the beginning of the contact ($x = 0$), the WS_2 strain appears to abruptly switch from tensile to compressive (**Figure S5a,b**). Under the contacts, the WS_2 is most compressive at the center of the contact, reaching a maximum of -0.15% compressive strain **(Figure S5a,b**). Outside the contact edges, the WS_2 strain is tensile then decays to "unstrained/neutral" away rom the contact edge.

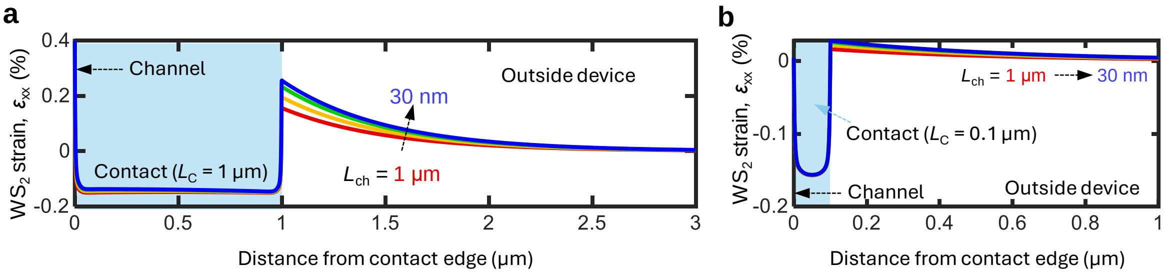

Figure S5. (a) Simulated horizontal strain profile along a device with $L_C = 1$ μm and $L_{ch} = 1$ μm, 300 nm, 100 nm, and 30 nm. The position x is normalized by L_{ch}, for easier visualization of different channel lengths. **(b)** Simulated horizontal strain profile along a device with $L_C = 0.1$ μm and $L_{ch} = 1$ μm, 300 nm, 100 nm, and 30 nm. **(c)** Simulated horizontal strain profile of WS_2 along a device with $L_C = 1$ μm and $L_{ch} = 1$ μm, 300 nm, 100 nm, and 30 nm from the inner contact edge at $x = 0$. **(d)** Simulated horizontal strain profile of WS_2 along a device with $L_C = 0.1$ μm and $L_{ch} = 1$ μm, 300 nm, 100 nm, and 30 nm from the inner contact edge at $x = 0$.

4. Photoluminescence Spectroscopy on WS_2

Monolayer WS_2 grown on sapphire was transferred onto thermally oxidized 100 nm SiO_2/Si and then patterned using electron-beam lithography. Ni/Au 15/20 nm was electron-beam evaporated for the contact features (as described in the Methods section). Spatially-resolved photoluminescence measurements of the WS_2 monolayer devices were obtained by confocal spectroscopy (Witec confocal Raman imaging microscope). To obtain the spatial map, the sample was exposed to a tightly focused 532 nm laser spot (×50 objective, NA = 0.55) using a high-resolution closed loop XYZ piezo scan stage. The excitation laser power was set to 126 μW and the photoluminescence signal was collected by the same objective lens and filtered by a dichroic filter cube and a long wave pass edge filter (Semrock, LP03-532RU-25).

To track the photoluminescence (PL) peaks, we fit the PL mapping spectra using an iterative least-square method in MATLAB. All PL spectra were taken from 1.78 eV to 2.2 eV and the baseline of the spectra were subtracted prior to fitting. Two peaks were used to fit the PL spectrum using a weighted Gaussian-Lorentzian line shape for all the peaks (**Figure S6a**). The higher energy (A exciton) peak was plotted for **Figure 2d,f** instead of the A^- lower energy shoulder peak. Both peaks were confirmed to show the same redshift with strain away from the contact, as well as the peak position for the combined PL peak (**Figure S6b**).

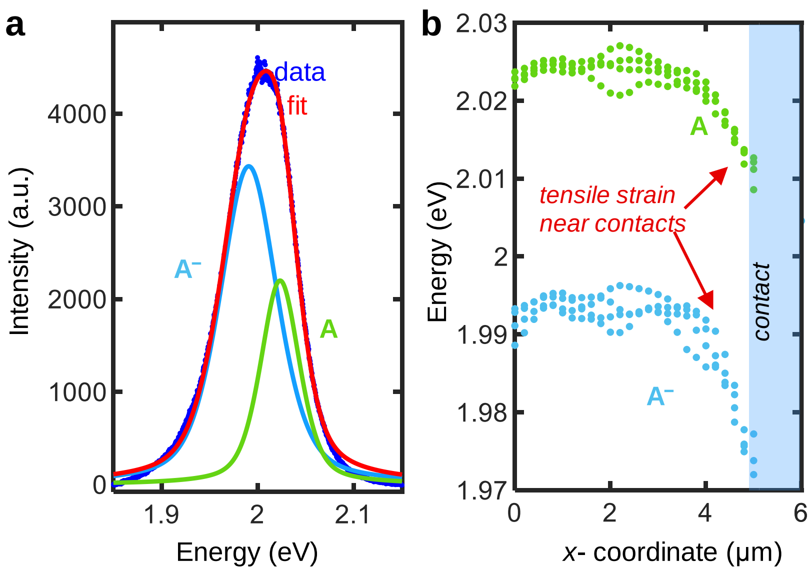

Figure S6. Photoluminescence measurements of WS_2 for strain determination. **(a)** PL spectra and peak fitting of A and A^- peaks. **(b)** Extracted WS_2 PL peak position of A and A^- peak position as a function of *x*-coordinate away from the contact edge (on the right). Plot uses data from **Figure 2d** and shows that both A and A^- peaks are redshifted near the contact, corresponding to tensile strain near the contact.

5. Literature Comparison for R_C in WS_2 and Annealing Conditions

Table S3: Benchmarking the electrical performance of WS_2 transistors.

Ref	WS_2 layers	Contact Metal	Annealed Contacts?	L_C (nm)	I_{on} (μA/μm)	L_{ch} (nm)	R_C (kΩ·μm)
**This Work**	**1L**	**Ni/Au (15/20 nm)**	**No**	**1 μm**	**223**	**50**	**1.73**
**This Work**	**1L**	**Ni/Au (15/20 nm)**	**No**	**0.1 μm**	**80**	**50**	**7.7**
15	1L	Au	No	Not reported	10	600	-
16	2L	Ni/Pd	Yes- 250 °C	Not reported	310	100	1.6
17	2L	Ni/Pd	Yes- 250 °C	Not reported	210	100	2.38
18	3-7L	Ni	385 K 6 h vacuum	Not reported	600	40	0.5
19	1L	Bi (20 nm)	No	Not reported	46	320	1.3
20	1L	Sb/Au (20/20)	No	~ 1 μm from SEM	243	135	0.73
20	1L	Bi/Au (20/20)	No	~ 1 μm from SEM	250	135	0.63
21	1L	Bi/Au (20/15 nm)	No	Not reported	400	100	-
22	1L	Ni/Au (20/40 nm)	No	~ 1 μm from SEM	325	50	1.1 (BeO)
22	1L	Ni/Au (20/40 nm)	No	~1 μm from SEM	170	50	2.6 (HfO_2)
23	1L	Ni (50 nm)	No	Not reported	150	290	1.2
24	2L	Ni/Au (20/40nm)	No	Not reported	635	18	0.38
24	1L	Ni/Au (20/40nm)	No	Not reported	267	80	0.72
25	2L (3R)	Ni/Au (20/60nm)	No	1 μm	480	50	0.67
26	1L	Ni/Au (40/30nm)	No	~ 1-1.3 μm from SEM	26	100	2.1
27	1L	Ni/Au (40/30nm)	No	Not reported	20	100	-

6. Transfer Length Determination for Ni Contacts on WS_2

The transfer length is given by $L_T = (\rho_C/R_{sh}')^{1/2}$, where R_{sh}' is the sheet resistance of the 2D channel under the contacts in Ω/□, and ρ_C is the specific contact resistivity given in Ω·μm^2. Contact resistance is can be expressed as $R_C = (\rho_C R_{sh}')^{1/2}\coth(L_C/L_T)$.

In the case when $L_C > 1.5\ L_T$ like in our L_C = 1 μm case, $R_C = (\rho_C R_{sh}')^{1/2}\coth(L_C/L_T) \approx (\rho_C R_{sh}')^{1/2}$. If we assume that $R_{sh}' = R_{sh}$ (the sheet resistance of WS_2 in the channel is the same as the resistance underneath the contacts), we can estimate the L_T of our Ni–WS_2 devices. For the L_C = 1 μm devices, fitting the median TLM devices at an overdrive voltage of V_{ov} = 1.95 V yields a R_C = 2.49 kΩ·μm and R_{sh} = 45 kΩ (**Figure S7a**). This would yield L_T = 63.5 nm, which is comparable to other results in 2D literature, in the 7–45 nm range.[28,29] When using the R_C extracted from the *best* TLM (1.7 kΩ·μm), the extracted L_T is 37 nm. Additionally, this extracted L_T could be considered the upper bound of the transfer length, since the R_{sh}' under the contacts would likely undergo contact deposition-induced damage, which could thus increase R_{sh}' and lower L_T. With L_T = 37 nm (63.5 nm), a L_C = 100 nm contact would increase in R_C due to current crowding at a maximum of 0.9% (8.9%). Thus, current crowding cannot solely explain the > 5× increase in contact resistance seen when L_C = 100 nm.

We also fabricate additional devices with L_C = 200 nm to confirm that our measured 100 nm devices are greater than the transfer length. We see that the L_C = 1 μm still shows significant improvement in on-state current than the L_C = 200 nm devices (**Figure S7b**).

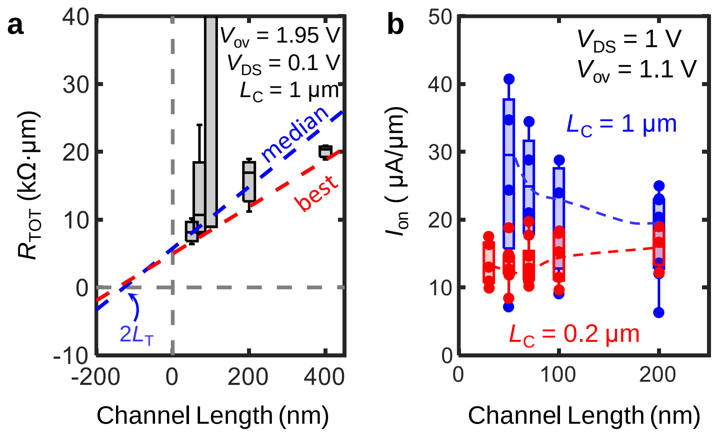

Figure S7. (a) Total device resistance R_{TOT} vs. channel length L_{ch}, at a fixed overdrive voltage V_{ov} = 1.1 V for a L_C = 1 μm device, showing linear fit of best devices (red) as well as median devices (blue). **(b)** On-state current (I_{on}) at a fixed overdrive V_{ov} = 1.1 V versus L_{ch}, comparing L_C = 1 μm and L_C = 0.2 μm, devices.

7. Additional Electrical Characterization and Analysis of Ni–WS_2 Devices on HfO_2

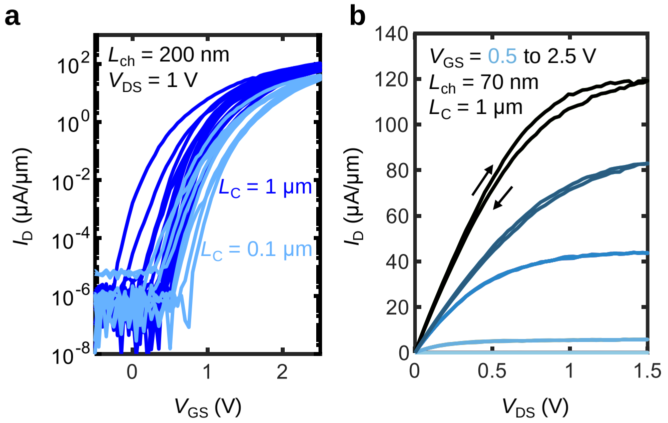

Figure S8. (a) Measured I_D vs. V_{GS} curves in log scale for all L_{ch} = 200 nm devices with L_C = 1 µm (8 devices) and L_C = 0.1 µm (4 devices). Forward and backward sweeps are plotted with clockwise hysteresis. **(b)** Measured I_D vs. V_{DS} curve for a representative L_{ch} = 70 nm device. Note the proper current saturation at relatively low voltage, due to the small, positive V_T.

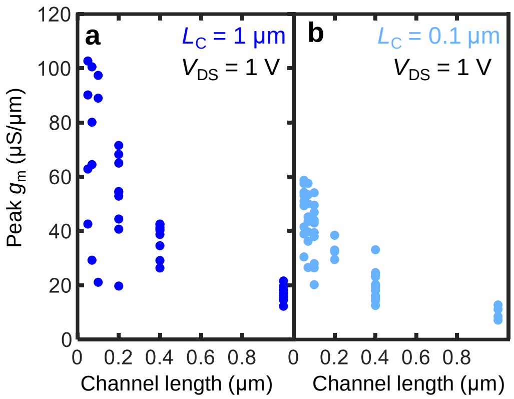

Figure S9. (a) Peak transconductance (g_m) vs. channel length for L_C = 1 µm. **(b)** Peak g_m vs. channel length for L_C = 0.1 µm.

Figure S10a compares the threshold voltage for long and short contacts, where most devices have a small, and positive V_T. For every channel length, the median V_T for long contact devices is more negative (**Figure S10a**). The boxplot in **Figure S10b** demonstrates that the long contact device has a median V_T = 0.56 V, compared to the short contact devices at V_T = 0.83 V. **Figure S10c** shows the hysteresis of the HfO_2 devices with various contact lengths plotted versus channel length. This was extracted at $I_D = 10^{-2}$ µA/µm.

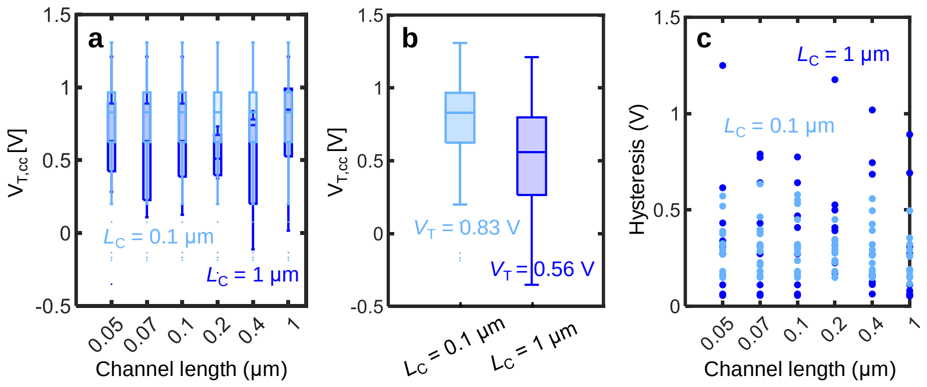

Figure S10. (a) Threshold voltage (V_T) extracted at $I_D = 10^{-2}$ μA/μm vs. channel length, for both long and short contact devices. (b) Boxplot of V_T for short and long contact devices, showing a more negative V_T for long contact devices. (c) Hysteresis at $I_D = 10^{-2}$ μA/μm with respect to channel length, for both long and short contacts, showing a much larger spread, and larger hysteresis for long contact devices.

8. Schottky Barrier Height Extraction

Additional devices were fabricated to confirm reproducibility of the strain effect of Ni contacts and to determine the Schottky barrier height (**Figure S11a**). Monolayer WS_2 was grown by CVD and then wet transferred onto 100 nm SiO_2 with pre-patterned alignment marks. The transfer process is described in the Methods Section. Coarse pads and channel definition were patterned and etched with XeF_2 into TLM structures with L_C = 1 μm and L_C = 0.1 μm. The fine contact step was patterned, then 15/20 nm Ni/Au were electron-beam deposited at ~ 10^{-8} Torr. The L_C = 1 μm devices showed roughly an order of magnitude increase in I_D at V_{GS} = 50 V, compared to the L_C = 0.1 μm devices (**Figure S11b**), as well as a negative threshold voltage shift for the long contact devices. This confirms the repeatability and generalization of our findings.

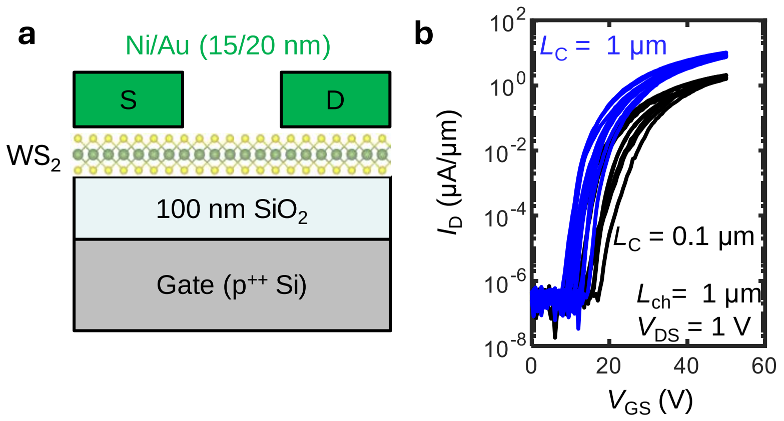

Figure S11. (a) Cross-section schematic of monolayer WS_2 transferred from sapphire onto 100 nm SiO_2 with electron-beam evaporated Ni/Au contacts. **(b)** Measured I_D vs. V_{GS} curves for L_{ch} = 1 μm devices with L_C = 1 μm (blue) and L_C = 0.1 μm (black), showing clear I_D improvement with long contacts. Both forward and backward sweeps are plotted, showing clockwise hysteresis.

For Schottky barrier height extraction, temperature-dependent measurements were conducted on 3 devices with L_C = 1 μm and 3 devices with L_C = 0.1 μm, all with L_{ch} = 1 μm. We estimate the Schottky barrier height (SBH, ϕ_B) from a procedure previously documented.[30] The thermionic emission current is given by $I_D = A_{2D}^* T^{\frac{3}{2}} \exp\left(-\frac{q\phi_B}{k_B T}\right)\left[1 - \exp\left(-\frac{qV}{k_B T}\right)\right]$, where A_{2D}^* is the 2D-equivalent Richardson constant, T is the temperature, k_B is Boltzmann's constant, q is the elementary charge, and V is the applied voltage. **Figure S12a,d** displays the transfer curves (I_D-V_{GS}) at each temperature, ranging from 193 K–313 K in steps of 20 K, for a L_C = 0.1 μm and L_C = 1 μm device. From the I_D-V_{GS} curves, an Arrhenius plot of $\ln(I_D/T^{\frac{3}{2}})$ vs $1000/T$ can be plotted for each voltage (V_{GS}) (**Figure S12b,e**), where the slope is the barrier height ϕ_B at that particular V_{GS}. The extracted barrier height is then plotted in **Figure S12c,f** for short and long contacts, respectively. The effective barrier height at the flat band voltage is estimated by the point at which the barrier height deviates from the linear fit (as seen in **Figure S12c,f**). For devices with L_C = 0.1 μm, barrier heights of 0.34 eV, 0.40 eV and 0.40 eV were extracted. For devices with L_C = 1 μm, barrier heights of 0.17 eV, 0.20 eV and 0.25 eV were extracted. On average, this corresponds to a barrier height lowering of 0.17 eV when switching to long contacts.

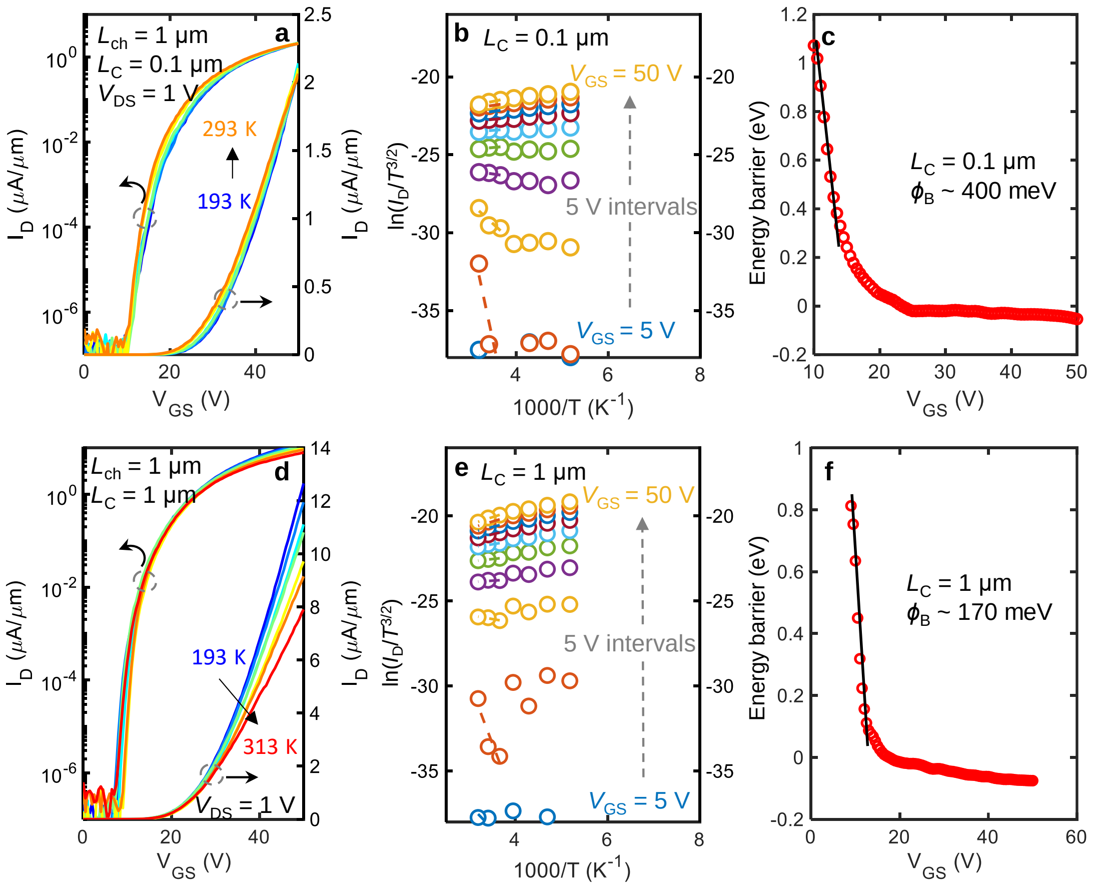

Figure S12. Schottky barrier height extraction of a typical L_{ch} = 1 μm Ni-WS_2 FETs on 100 nm SiO_2. **(a-c)** L_C = 0.1 μm and **(d-f)** L_C = 1 μm. **(a), (d)** Temperature-dependent I_D–V_{GS} measured at V_{DS} = 1 V. **(b), (e)** Arrhenius plots of Ni–WS_2 FETs for V_{GS} from 5 V to 50 V. **(c), (f)** Extracted electron Schottky barrier height.

9. Utilizing Capping Layers for Thermal Processing

Capping layers can be used to pin the WS_2 channel during thermal annealing, in order to maintain the strain profile in a device. Here, 1.5 nm Al was electron-beam evaporated on the HfO_2 local back-gate devices. Then, 10 nm AlO_x was deposited by thermal atomic layer deposition at 130°C (**Figure S13a**). The devices were measured before capping, after capping, and after a 150°C 2 hour vacuum anneal. The long contacts devices did not show any degradation in performance, even showing an increase in I_D (**Figure S13b**). In capped devices, the WS_2 is less likely to slip from the substrate. The top oxide layer can "pin down" the WS_2 in place. This contrasts the uncapped devices, which showed lower I_D after annealing (**Figure 4a,c**). The short contact devices did not show a significant change in performance after annealing **(Figure S13c**). Future work is needed to determine how the strain profile is influenced by encapsulation, thermal processing, and different device geometries (e.g. gate-all-around, dual-gate structures), for industry relevant processing.

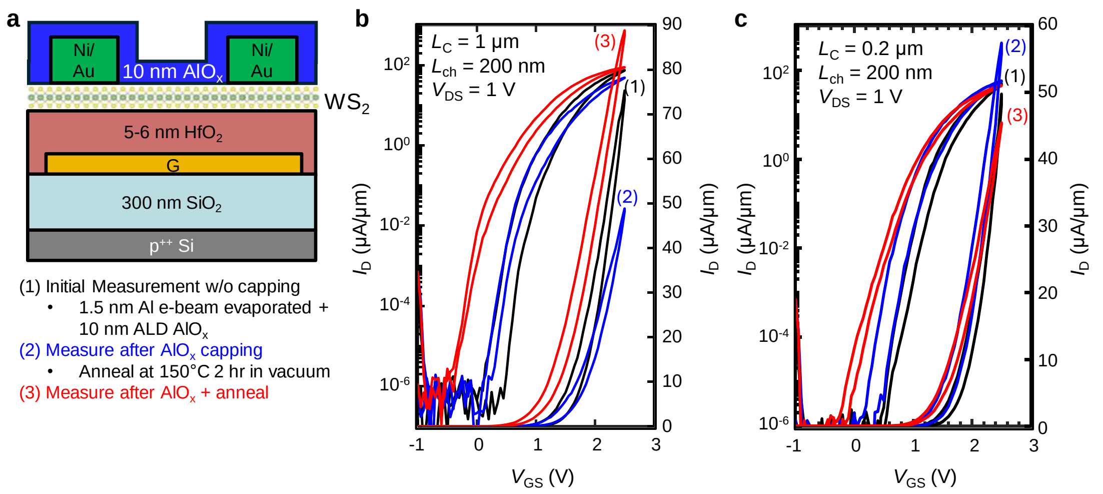

Figure S13. **(a)** Device schematic of a local back-gated monolayer WS_2 transistor after AlO_x capping (top) and device fabrication process with device measurement steps after device fabrication (bottom). Stages (1) - (3) are denoted for panels (b,c) **(b)** Measured I_D vs. V_{GS} for a $L_C = 1$ μm device with $L_{ch} =$ 200 nm. The 3 stages of the single device are plotted (as given by panel (a), bottom). **(c)** Measured I_D vs. V_{GS} for a $L_C = 0.2$ μm device with $L_{ch} = 200$ nm. All measurements are taken at room temperature at ~10^{-4} Torr vacuum probe station.

10. Strain Evaluation of Contact Metal Thin Films via X-ray Diffraction

We quantified changes in the structure and stress state of the Ni/Au films using in-plane X-ray diffraction. Continuous monolayer WS_2 was transferred from sapphire onto SiO_2/Si. To mimic the make-up of our WS_2 contacts, 15/20 nm Ni/Au was then blanket-deposited on top. X-ray diffraction measurements were conducted using a PANalytic Empyrean system with a Cu-Kα target. All in-plane measurements were conducted with χ (as defined previously[31]) at 88.5°– 89.25° to measure the in-plane diffraction peaks at various diffraction angles (2θ). To measure the changes in the Ni in-plane strain, only 15 nm of Ni was deposited: due to the shallow angle of the X-ray beam, the X-rays were all absorbed in the top Au layer. Symmetric $\theta/2\theta$ scans were used to verify the planes present in the films. We separately measured the changes in Ni strain on fused silica (amorphous SiO_2) to confirm that the nearby crystalline Si diffraction peaks were not convolved in our measurement.

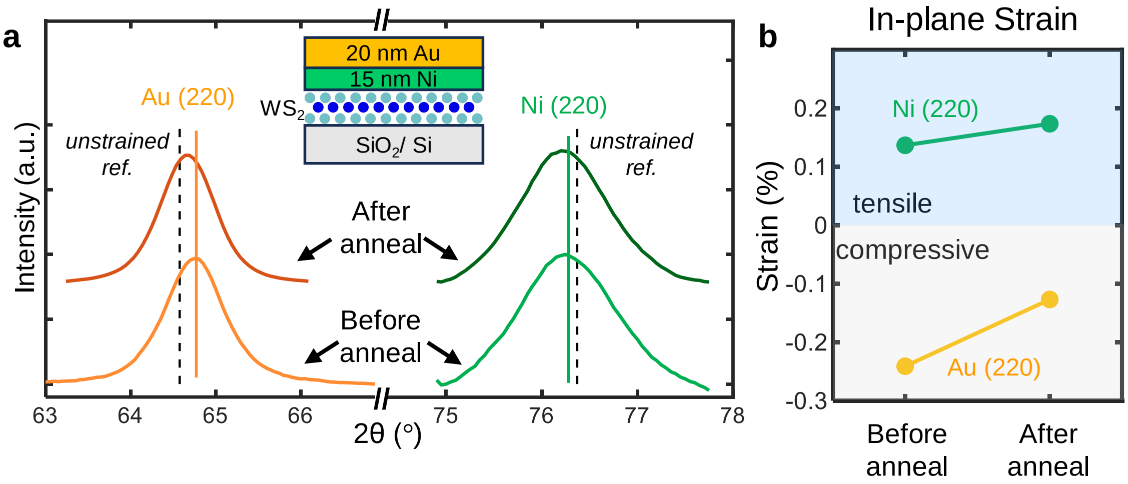

Figure S14. X-ray diffraction for in-plane strain in metal films. **(a)** In-plane XRD spectra of the Au (220) and Ni (220) peaks collected before and after annealing at 150 °C. **(b)** Calculated in-plane strain in the Ni and Au layers before and after annealing.

Ni was found to evaporate with a high degree of in-plane tensile strain (0.137%), while Au is evaporated with some compressive strain (**Figure S14a,b**). The high degree of tensile in-plane strain in the Ni combined with its high Young's modulus (~200 GPa) is responsible for the substantial strain induced in the WS_2 contact region, which is much more mechanically compliant than the Au film.

After annealing, we observe that the in-plane strain of both contact metals becomes more tensile, from 0.137% to 0.174% for Ni and from -0.241% to -0.127% for Au (**Figure S14a,b**). Annealing is seen to more dramatically affect the Au layer, which becomes much more tensile in-plane. This is expected as Au has a higher thermal expansion coefficient than Ni. There may also be additional transient strains due to differences in thermal expansion coefficients that impact the strain in Ni, Au, and WS_2. One could manipulate the thermal response by carefully considering a metal's oxidation potential and its thermal response, as well as the impacts of a bilayer metal stack. One way to overcome the effect of annealing is to potentially use a low expansion contact such as iron-nickel alloys.[32]

From initial in-plane measurements on the SiO_2/Si substrate, the Ni (220) peak was determined to be in-plane oriented. However, the Ni (220) closely aligns to the Si (331) peak. To reaffirm that the peaks measured were from Ni, continuous monolayer WS_2 was also transferred onto fused silica. Again, 15 nm Ni was blanket deposited onto the continuous monolayer WS_2 by electron beam evaporation. **Figure S15** validates that 1) the in-plane peak is once again Ni (220), and that 2) the peak positions and thus strain of the Ni film deposited on both fused silica and SiO_2/Si are the same.

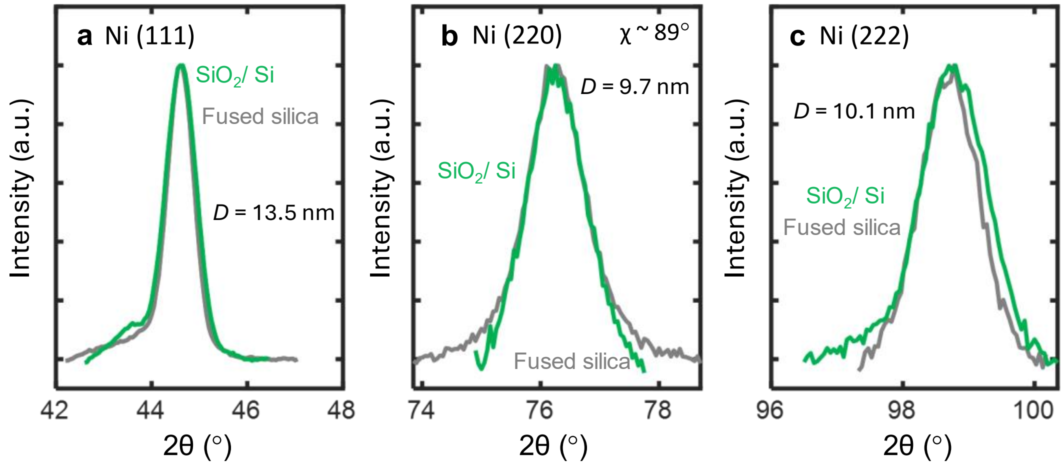

Figure S15. X-ray diffraction on Ni film blanket deposited on SiO_2/Si (green) and fused silica (grey). **(a)** Ni (111) peak. **(b)** Ni (220) peak. **(c)** Ni (222) peak. *D* is the average grain size, calculated from the Scherrer equation.

We note that decoupling grain size from stress effects is difficult. For polycrystalline metal films that grow in the Volmer-Weber mode (like Ni), it is understood that stresses developed during deposition are related to the morphologies and microstructures of the growing films. The development of a tensile coalescence stress is understood to be from grain boundary formation and thus grain size and stress are linked.[33]

Evaporated Ni metal grains are typically on the order of a few nanometers (<5 nm) initially, which increases to 20 nm in a 70 nm-thick Ni film.[34] Using the Scherrer equation for the XRD spectra in **Figure S15**, the average grain size is 13.53 nm, 9.70 nm, 10.10 nm, for the spectra obtained for the Ni (111), (220), and (222) on fused silica, respectively. This is an order of magnitude lower than the shortest contact length (100 nm) used. Thus, we don't expect the grain size to vary significantly between the two contact lengths.

11. Effect of Annealing on Ni–WS_2 Devices on SiO_2

Ni-contacted monolayer WS_2 devices were fabricated on 100 nm SiO_2 as described in **Supporting Information Section 1** and **Figure S1**, on top of WS_2 directly grown on the SiO_2. 20/35 nm Ni/Au was used as the contacts, with device width W = 2 µm and L_C = 1.5 µm. **Figure S16** displays the transfer curves of the devices for L_{ch} = 100 nm–1 µm. Initially, there was low yield of the L_{ch} = 100 nm devices, probably due to the stress imparted by the contact stack for short channel dimensions, causing cracking. After a 250°C 2 hour vacuum anneal, the same trend was seen as the 150°C anneal for long contact devices, where the I_{on} and R_C worsened with annealing.

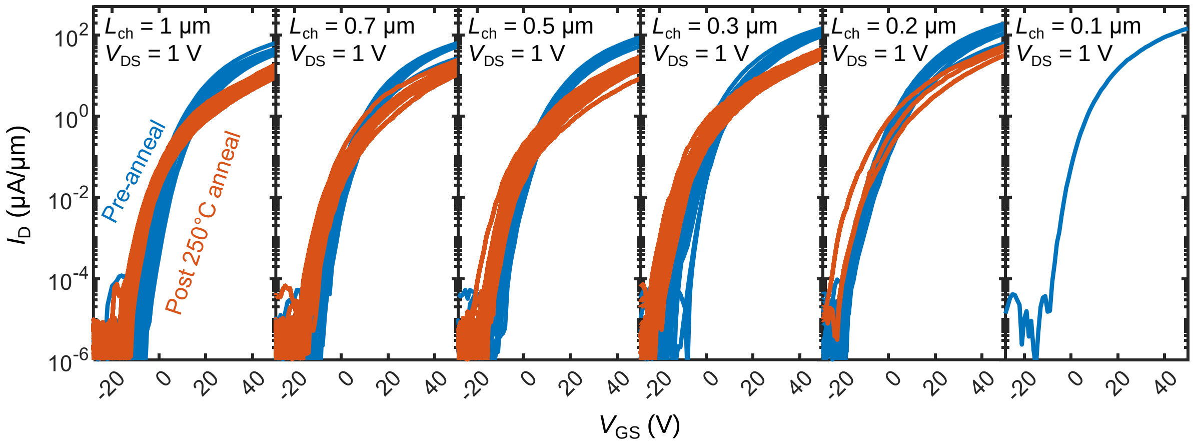

Figure S16. Transfer curves of working devices for Ni contacts on monolayer WS_2 with a 100 nm SiO_2 gate dielectric (L_{ch} = 100 nm–1 µm, W = 2 µm and L_C = 1.5 um), for before and after vacuum annealing at 250°C. Only forward sweep has been plotted for clarity.

After the 250 °C anneal, very few of the L_{ch} = 200 nm devices worked, and the majority of the devices were open circuit when measured. In comparison, all the long channel devices (L_{ch} = 0.7-1 µm) that worked pre-anneal, worked after annealing (**Figure S17a**). The devices were examined using scanning electron microscopy (SEM) to find the root cause of failure. For the 200 nm and 300 nm devices that worked before annealing but not after annealing, a crack across the channel was seen in the WS_2 (**Figure S17b**). The evidence of cracking as well as the likelihood to affect shorter channels, illustrates the high stress in the WS_2 especially for the short channel devices. Additionally, the cracking arising specifically from annealing demonstrates the high stress WS_2 experiences during annealing with Ni/Au contacts. This is supported by the XRD results in **Supporting Information Section 10** that indicates increased tensile strain in both the Ni and Au with annealing.

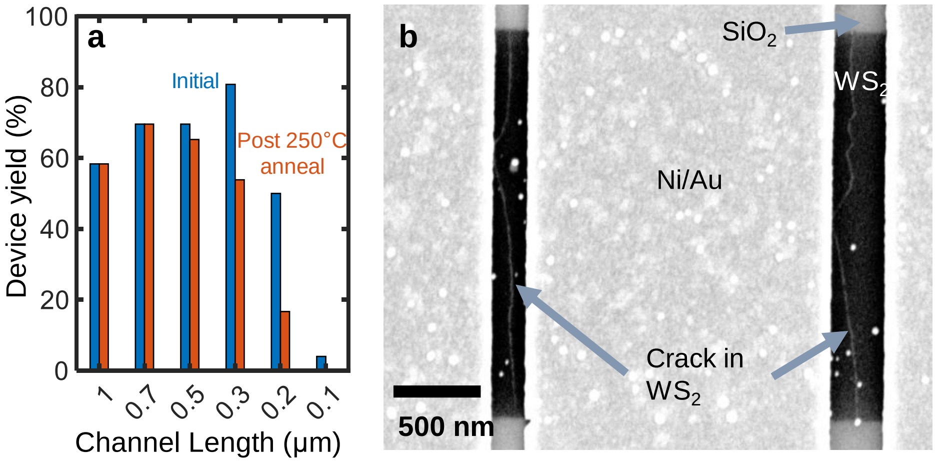

Figure S17. (a) The number of working devices at each channel length, before and after annealing, for devices shown in **Figure S16. (b)** Scanning electron microscopy (SEM) image of a Ni/Au 20/35 nm device, showing cracking in both the L_{ch} = 200 nm (left) and L_{ch} = 300 nm (right) after undergoing a 250 °C vacuum anneal.

12. Density Functional Theory Simulations

Density functional theory (DFT) simulations were performed using Quantum ESPRESSO version 7.1.[35] We use spin-orbit coupling and fully relativistic norm-conserving Vanderbilt pseudopotentials.[36,37] The energy band diagrams were extracted as functions of biaxial and uniaxial strain, similar to our previous work,[38] and corresponding band gaps were determined (**Figure S18a**). Biaxial and uniaxial tensile strain both lead to a band gap reduction, mainly through the lowering of the conduction band edge at the K point. Here, we extract both the movement of the conduction band edge and valence band edge with respect to strain, showing that tensile strain highly impacts the movement of the conduction band edge at a much faster rate than the valence band edge (**Figure S18b**). DFT calculations suggest that an average reduction of 170 meV in conduction band minimum (i.e., barrier height, **Figure 3f),** corresponds to ~1.5% uniaxial strain induced in the WS_2 near the contacts. This is in reasonable agreement with the values obtained from finite-element analysis simulations (~0.2–0.6%) and photoluminescence (~0.7%) (**Figure 2f**).

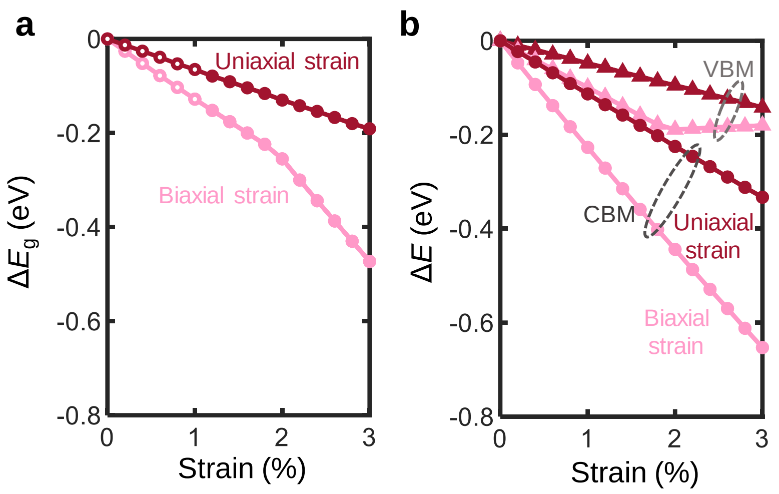

Figure S18. (a) Calculated energy band gap reduction (ΔE_g) for uniaxial and biaxial tensile strain. Hollow circles denote data from Yang et al.[33] **(b)** Change in energy of conduction band minimum (CBM) and valence band maximum (VBM) under uniaxial tensile strain (dark red) and biaxial tensile strain (pink).

13. Finite Element Analysis Simulations with Varying Ni Thickness

The strain profile of WS_2 was also simulated with different Ni contact thicknesses (**Figure S19**) of 5 nm and 50 nm (in addition to the 15 nm in all other simulations), with a fixed film stress of 400 MPa. The strain in the TMD has been shown to be proportional to the thin film force.[7,39,40] **Figure S20** shows that as the Ni thickness (and thus film force) increases, the tensile strain in the WS_2 channel increases.

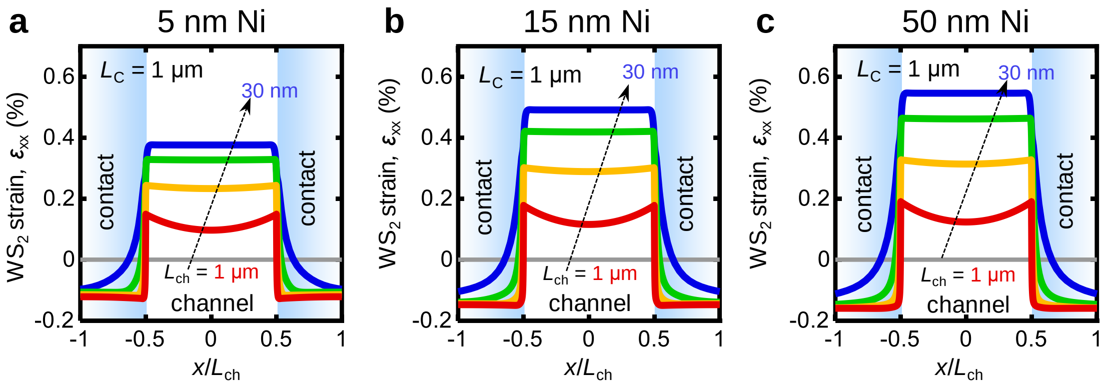

Figure S19. Simulated horizontal strain profile along a device with L_C = 1 µm and L_{ch} = 1 µm, 300 nm, 100 nm, and 30 nm with Ni contact thickness of **(a)** 5 nm **(b)** 15 nm **(c)** 50 nm. The position x is normalized by L_{ch}, for easier visualization of different channel lengths.

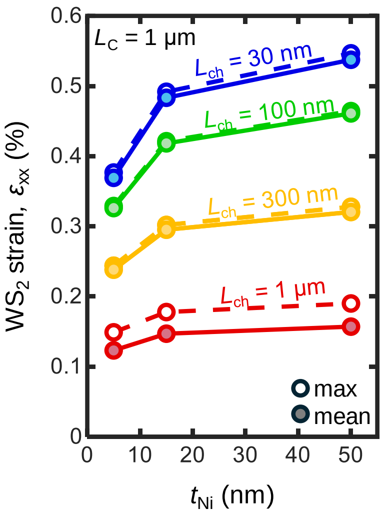

Figure S20: Simulated horizontal strain of WS_2 as a function of Ni contact thickness for L_C = 1 µm and L_{ch} = 1 µm, 300 nm, 100 nm, and 30 nm. Filled circles denote the mean strain, open circles denote the maximum strain value in the channel.

REFERENCFES FOR SI

1. English, C. D., Shine, G., Dorgan, V. E., Saraswat, K. C. & Pop, E. Improved Contacts to MoS_2 Transistors by Ultra-High Vacuum Metal Deposition. *Nano Lett.* **16**, 3824–3830 (2016).
2. Chou, A.-S. *et al.* Antimony Semimetal Contact with Enhanced Thermal Stability for High Performance 2D Electronics. in *2021 IEEE International Electron Devices Meeting (IEDM)* 7.2.1-7.2.4 (IEEE, 2021). doi:10.1109/IEDM19574.2021.9720608.
3. Janssen, G. C. A. M., Abdalla, M. M., van Keulen, F., Pujada, B. R. & van Venrooy, B. Celebrating the 100th anniversary of the Stoney equation for film stress: Developments from polycrystalline steel strips to single crystal silicon wafers. *Thin Solid Films* **517**, 1858–1867 (2009).
4. Li, L., Zeng, Z. Y., Liang, T., Tang, M. & Cheng, Y. Elastic properties and electronic structure of WS_2 under pressure from first-principles calculations. *Z. Naturforsch. A* **72**, 295–301 (2017).
5. Vargas, A. L. M., De Araújo Ribeiro, F. & Hübler, R. Changes in the Young Modulus of hafnium oxide thin films. *Nucl. Instrum. Methods Phys. Res., Sect. B* **365**, 362–366 (2015).
6. Berdova, M. *et al.* Hardness, elastic modulus, and wear resistance of hafnium oxide-based films grown by atomic layer deposition. *J. Vac. Sci. Technol. A* **34**, 051510 (2016).
7. Zhang, Y. *et al.* Patternable Process-Induced Strain in 2D Monolayers and Heterobilayers. *ACS Nano* **18**, 4205–4215 (2024).
8. Gong, L. *et al.* Interfacial stress transfer in a graphene monolayer nanocomposite. *Adv. Mater.* **22**, 2694–2697 (2010).
9. Guo, G. & Zhu, Y. Cohesive-Shear-Lag Modeling of Interfacial Stress Transfer between a Monolayer Graphene and a Polymer Substrate. *J. Appl. Mech.* **82**, 031005 (2015).
10. Cox, H. L. The elasticity and strength of paper and other fibrous materials. *Br. J. Appl. Phys.* **3**, 72–79 (1952).
11. Bronsgeest, M. S. *et al.* Strain Relaxation in CVD Graphene: Wrinkling with Shear Lag. *Nano Lett.* **15**, 5098–5104 (2015).
12. Wang, F. *et al.* Strain engineering in monolayer WS_2 and WS_2 nanocomposites. *2D Mater.* **7**, 045022 (2020).
13. Jiang, T., Huang, R. & Zhu, Y. Interfacial sliding and buckling of monolayer graphene on a stretchable substrate. *Adv. Funct. Mater.* **24**, 396–402 (2014).
14. Dai, Z., Lu, N., Liechti, K. M. & Huang, R. Mechanics at the interfaces of 2D materials: Challenges and opportunities. *Curr. Opin. Solid State Mater. Sci.* **24**, 100837 (2020).
15. Dorow, C. *et al.* Advancing Monolayer 2-D nMOS and pMOS Transistor Integration from Growth to Van der Waals Interface Engineering for Ultimate CMOS Scaling. *IEEE Trans. Electron Devices* **68**, 6592–6598 (2021).
16. Lin, D. *et al.* Scaling synthetic WS_2 dual-gate MOS devices towards sub-nm CET. in *2021 Symposium on VLSI Technology* vol. 1 1–2 (2021).
17. Lin, D. *et al.* Dual gate synthetic WS_2 MOSFETs with 120μS/μm Gm 2.7μF/cm^2 capacitance and ambipolar channel. in *2020 IEEE International Electron Devices Meeting (IEDM)* 3.6.1-3.6.4 (IEEE, 2020). doi:10.1109/IEDM13553.2020.9372055.
18. Pang, C. S., Wu, P., Appenzeller, J. & Chen, Z. Thickness-Dependent Study of High-Performance WS_2-FETs with Ultrascaled Channel Lengths. *IEEE Trans. Electron Devices* **68**, 2123–2129 (2021).

19. Jin, L. & Koester, S. J. Contact Gating in Dual-Gated WS_2 MOSFETs with Semi-Metallic Bi Contacts. *IEEE Electron Device Lett.* **43**, 1575–1578 (2022).
20. Li, M.-Y. *et al.* Wafer-Scale Bi-Assisted Semi-Auto Dry Transfer and Fabrication of High-Performance Monolayer CVD WS_2 Transistor. in *2022 IEEE Symposium on VLSI Technology and Circuits (VLSI Technology and Circuits)* 290–291 (IEEE, 2022).
21. Wan, Y. *et al.* Low-defect-density WS_2 by hydroxide vapor phase deposition. *Nat. Commun.* **13**, 4149 (2022).
22. Shi, X. *et al.* Improved Self-Heating in Short-Channel Monolayer WS_2 Transistors with High-Thermal Conductivity BeO Dielectrics. *Nano Lett.* **22**, 7667–7673 (2022).
23. Sun, Z. *et al.* Statistical Assessment of High-Performance Scaled Double-Gate Transistors from Monolayer WS_2. *ACS Nano* **16**, 14942–14950 (2022).
24. Shi, X. *et al.* Ultrashort channel chemical vapor deposited bilayer WS_2 field-effect transistors. *Appl. Phys. Rev.* **10**, 011405 (2023).
25. Li, X. *et al.* Rhombohedral-stacked bilayer transition metal dichalcogenides for high-performance atomically thin CMOS devices. *Sci. Adv.* **9**, eade5706 (2023).
26. Sebastian, A., Pendurthi, R., Choudhury, T. H., Redwing, J. M. & Das, S. Benchmarking monolayer MoS_2 and WS_2 field-effect transistors. *Nat. Commun.* **12**, 693 (2021).
27. Chubarov, M. *et al.* Wafer-Scale Epitaxial Growth of Unidirectional WS_2 Monolayers on Sapphire. *ACS Nano* **15**, 2532–2541 (2021).
28. Shen, P.-C. *et al.* Ultralow contact resistance between semimetal and monolayer semiconductors. *Nature* **593**, 211–217 (2021).
29. Schranghamer, T. F. *et al.* Ultrascaled Contacts to Monolayer MoS_2 Field Effect Transistors. *Nano Lett.* **23**, 3426–3434 (2023).
30. Das, S., Chen, H. Y., Penumatcha, A. V. & Appenzeller, J. High performance multilayer MoS_2 transistors with scandium contacts. *Nano Lett.* **13**, 100–105 (2013).
31. Fitzpatrick, M. E., Fry, A. T. & Holdway, P. *Determination of residual stresses by x-ray diffraction*. (2005).
32. Cverna, F. *ASM ready reference: thermal properties of metals*. vol. 831 (ASM International, 1998).
33. Nix, W. D. Metallic thin films: Stresses and mechanical properties. in *Metallic Films for Electronic, Optical and Magnetic Applications.* 353–421 (Woodhead Publishing, 2014).
34. Yu, H. Z. & Thompson, C. V. Grain growth and complex stress evolution during Volmer-Weber growth of polycrystalline thin films. *Acta Mater.* **67**, 189–198 (2014).
35. Giannozzi, P. *et al.* Quantum ESPRESSO toward the exascale. *J. Chem. Phys.* **152**, 154105 (2020).
36. Schlipf, M. & Gygi, F. Optimization algorithm for the generation of ONCV pseudopotentials. *Comput. Phys. Commun.* **196**, 36–44 (2015).
37. Scherpelz, P., Govoni, M., Hamada, I. & Galli, G. Implementation and Validation of Fully Relativistic GW Calculations: Spin-Orbit Coupling in Molecules, Nanocrystals, and Solids. *J. Chem. Theory Comput.* **12**, 3523–3544 (2016).
38. Yang, J. A. *et al.* Biaxial Tensile Strain Enhances Electron Mobility of Monolayer Transition Metal Dichalcogenides. *ACS Nano* **18**, 18151–18159 (2024).
39. Peña, T. *et al.* Strain engineering 2D MoS_2 with thin film stress capping layers. *2D Mater.* **8**, 045001 (2021).
40. Jaikissoon, M., Pop, E. & Saraswat, K. C. Strain Induced by Evaporated-Metal Contacts on Monolayer MoS_2 Transistors. *IEEE Electron Device Lett.* **45**, 1528–1531 (2024).
